# Multiple reaction pathway on alkaline earth imide supported catalysts for efficient ammonia synthesis

**DOI:** 10.1038/s41467-023-42050-7

**Published:** 2023-10-11

**Authors:** Zichuang Li, Yangfan Lu, Jiang Li, Miao Xu, Yanpeng Qi, Sang-Won Park, Masaaki Kitano, Hideo Hosono, Jie-Sheng Chen, Tian-Nan Ye

**Affiliations:** 1 Frontiers Science Center for Transformative Molecules, School of Chemistry and Chemical Engineering, Shanghai Jiao Tong University, Shanghai, 200240 China; 2 College of Materials Science and Engineering, National Engineering Research Center for Magnesium Alloys, Chongqing University, Chongqing, 400044 China; 3 Materials Research Center for Element Strategy, Tokyo Institute of Technology, 4259 Nagatsuta, Midori-ku, Yokohama, 226-8503 Japan; 4 State Key Laboratory of Space Power Sources, Shanghai Institute of Space Power-Sources, Shanghai, 200245 China; 5 School of Physical Science and Technology Shanghai Tech University, Shanghai, 201210 China; 6 ShanghaiTech Laboratory for Topological Physics, ShanghaiTech University, Shanghai, 201210 China; 7 Shanghai Key Laboratory of High-resolution Electron Microscopy, ShanghaiTech University, Shanghai, 201210 China

**Keywords:** Heterogeneous catalysis, Materials for energy and catalysis

## Abstract

The tunability of reaction pathways is required for exploring efficient and low cost catalysts for ammonia synthesis. There is an obstacle by the limitations arising from scaling relation for this purpose. Here, we demonstrate that the alkali earth imides (*Ae*NH) combined with transition metal (TM = Fe, Co and Ni) catalysts can overcome this difficulty by utilizing functionalities arising from concerted role of active defects on the support surface and loaded transition metals. These catalysts enable ammonia production through multiple reaction pathways. The reaction rate of Co/SrNH is as high as 1686.7 mmol·g_Co_^−1^·h^−1^ and the TOFs reaches above 500 h^−1^ at 400 °C and 0.9 MPa, outperforming other reported Co-based catalysts as well as the benchmark Cs-Ru/MgO catalyst and industrial wüstite-based Fe catalyst under the same reaction conditions. Experimental and theoretical results show that the synergistic effect of nitrogen affinity of 3d TMs and in-situ formed NH^2−^ vacancy of alkali earth imides regulate the reaction pathways of the ammonia production, resulting in distinct catalytic performance different from 3d TMs. It was thus demonstrated that the appropriate combination of metal and support is essential for controlling the reaction pathway and realizing highly active and low cost catalysts for ammonia synthesis.

## Abstract (web summary)

The presence of electrically active defects on the surface of the support has been shown to be effective for N_2_ activation. Here the authors discover that electron-rich polyanionic NH^2−^ defect allows for efficient ammonia synthesis via multiple reaction pathway by incorporating various affordable transition metals.

## Introduction

Ammonia (NH_3_) has been one of the most critical intermediates for various chemicals and fertilizers^1,2^. Recently, NH_3_ has also attracted attention as a renewable energy carrier because of its high energy density (22.5 kJ‧g^−1^) and hydrogen content (17.6 wt%)^3,4^. The industrial NH_3_ is majorly produced by Haber–Bosch process that requires high temperature (400–600 °C) and pressure (20–40 MPa) conditions^5^. In ammonia synthesis, the dissociation of nitrogen molecules is regarded as the most challenging step due to its stable N≡N triple bond^6–8^. Therefore, many of the previous studies have focused on weakening the N≡N bond during catalytic reactions. For example, in Fe and Ru-based catalysts, the addition of basic promoters, such as alkali and alkaline earth metal oxides, have been intensively investigated because they give rise to electron transfer from the catalysts to the anti-bonding (π*) state of nitrogen molecules, weakening the N≡N bond through electron donation mechanism^9–18^. However, the promotion effect of basic supports is not sufficient to facilitate ammonia synthesis under mild conditions due to the limited electron donation ability. Another approach to lower the apparent activation energy is to use electride as support materials^19–29^, such as the 12CaO·7Al_2_O_3_ (C12A7:e^−^) electride. In contrast to the traditional promoters, C12A7:e^−^ is characterized by the co-existence of high electron density with low work function, chemical and thermal stability, and reversible exchangeability between anionic electron and hydrogen, realizing much stronger electron transfer from support to active transition metals and robustness to hydrogen poisoning. Consequently, the rate-determine step of ammonia synthesis was shifted from N_2_ dissociation to the NH_*x*_ formation in Ru/C12A7:e^−^, contributing to suppress the reaction activation energy^30^.

In either traditional or electride-based catalysts, their transition metal sites are responsible for catalytic reactions, and the catalytic activity strongly depends on the magnitude of the interaction between nitrogen and transition metals, which is widely known as the scaling relation^31^. In this reaction mechanism, Ru is located at around the optimal point and thus show excellent catalytic activity, while low cost 3d transition metals, such as Fe, Co and Ni, were less effective under mild reaction condition^32–38^. Recently, the combination of hydride and transition metals were focused on overcoming the bottlenecks. Chen et al.^39,40^ showed that the scaling relation, as well as the reaction path of ammonia synthesis, were shifted by using the transition metals loaded LiH (TM/LiH), in which N_2_ molecule initially dissociated to N* on the surface of TMs and then transferred to LiH support, forming the Li-NH_*x*_ species as intermediates. Subsequently, the NH_x_ species undergo further hydrogenation to form NH_3_. Such reaction path has circumvented the limitation of the scaling relation by functionalizing the lattice hydrogen of LiH supports. Similar strategy was also proved to be effective on Fe/TiO_2−*x*_H_*y*_ catalyst^41^, in which N_2_ and H_2_ are both dissociated on Fe and reacted with each other to form NH_3_ in the oxygen vacancy site through a spillover process. Recently, transition metal nitrides, especially Co_3_Mo_3_N, have also received great attention for ammonia synthesis since the nitrogen defects are reported to be effective for the nitrogen molecule activation through a Mars-van Krevelen mechanism, which can significantly weaken the N≡N bond and lower its dissociation barrier^42–46^. For each case, the modified reaction path from TMs to support material leads to a low activation energy barrier, allowing NH_3_ synthesis at low temperatures.

Based on a concept of the synergistic effect between TMs and supports’ defects, we developed TM/ReN (TMs = Co and Ni; Re = La and Ce) catalysts for ammonia synthesis^47–49^. Among them, the dual active site strategy was utilized, enabling the N_2_ activation at the nitrogen vacancy (V_N_) sites of ReN support. That is, V_N_ has trapped electrons with low work function reflecting the property of rare earth. This is the reason why V_N_ in ReN works well as sites for N_2_ activation. Ni/ReN successfully overcame the scaling limitations associated with the weak nitrogen binding energy of Ni. In Co/CeN, the in situ formed V_N_ sites provide sites for N_2_-activation as well. V_N_ in ReN is an anionic defect and featured by electron-rich and low-work function properties^47^. These results inspire us to extend this idea to other system; the electron-rich vacancies would be effective in improving the catalytic activity of 3d TMs themselves that facilitate dissociative N_2_ adsorption aside from defects driven pathway, giving rise to the associative-dissociative concerted mechanism. So far, most of the defect-driven ammonia synthesis catalysts utilize anionic defects of single atoms, such as O^2−^, H^−^ and N^3−^^41,50,51^, while the functionalities of polyanions, such as NH^2−^, remained unexplored despite of their intriguing properties associated with its large anionic volume that would be favorable for N_2_ activation with expectation for allowing various adsorption geometry for adsorbed N_2_ and enhanced trapping efficiency of lower work function electrons. We are therefore motivated to explore the materials having larger defect sites, enabling to control the reaction pathway of ammonia synthesis and further improve its catalytic activity.

Here, we report that alkaline earth imides, *Ae*NH (*Ae* = Ca, Sr, Ba), is one of the ideal platforms to control the reaction pathway by utilizing its anionic defects. In contrast to the previously studied materials, the anionic defects of *Ae*NH are composed of polyanionic group (NH^2–^), which offer much larger space to host nitrogen, i.e., the NH^2–^ defect is 1.2 times as large as N^3–^ defect in LaN. DFT calculation shows that defective SrNH has much lower work function property than previously studied CeN and LaN. As the results, the nitrogen adsorption properties of TMs are strongly affected by the in situ formed NH^2−^ vacancy of *Ae*NH, enabling ammonia production through multiple reaction pathways. By loading 3d TMs (TM = Fe, Co and Ni), TM/*Ae*NH continuously produced ammonia and reached 779.2 mmol·g_Co_^−1^·h^−1^ at 400 °C and 0.9 MPa for Co/SrNH, which is six times greater than previously studied Ni/LaN. Isotope experiments in combination with DFT calculations clarifies that the cooperation of the surface low work function (~2.0 eV) feature and the in situ formation of large sized NH^2−^ vacancies on SrNH support gives rise to a dual pathway for ammonia synthesis over Co/SrNH catalyst. These discoveries show the introduction of large sized electron-rich anionic vacancy enables N_2_ activation with low activation energy barrier, providing a material design strategy to realize highly efficient ammonia synthesis under mild reaction conditions.

## Results

Density functional theory (DFT) calculations were first performed to investigate the NH^2−^ vacancy formation of the *Ae*NH (*Ae* = Ca, Sr, Ba) (Fig. S1a). The calculated vacancy formation energy (E_V_) for CaNH, SrNH, and BaNH are estimated to be BaNH (0.85 eV) < SrNH (1.92 eV) < CaNH (2.35 eV), indicating the formation of NH^2−^ vacancy is more energy favored for BaNH and SrNH comparing to CaNH. Notably, E_V_ of SrNH and BaNH are comparable to those of LaN (1.90 eV) and CeN (1.39 eV). The Bader charge of the V_NH_ sites in CaNH, SrNH, and BaNH were calculated to be −1.24, −1.54 and −1.61 (Fig. S1b–d), respectively, which suggested that substantial electrons originated from the NH^2−^ ion are accumulated in V_NH_ sites of *Ae*NH^52^, indicating that the defects also exhibit strong electron donation ability. Interestingly, *Ae*NH with surface V_NH_ sites was found to show extremely low work function characteristics, which will be discussed below. We thus expect the easily formed electron-rich NH^2−^ vacancy site can provide additional active sites for N_2_ activation and promote a high catalytic performance in ammonia synthesis.

**Fig. 1:**
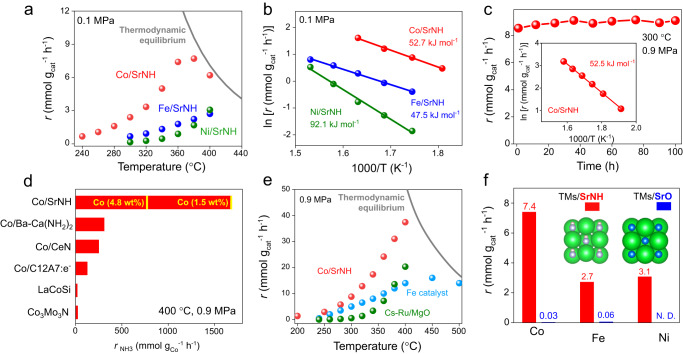
Catalytic activity of *TM*/*Ae*NH catalysts. **a** Temperature dependence of the NH_3_ synthesis rates over Co/SrNH, Fe/SrNH and Ni/SrNH catalysts under 0.1 MPa. **b** Arrhenius plots for NH_3_ synthesis over Co/SrNH, Fe/SrNH and Ni/SrNH catalysts at 0.1 MPa. **c** Stability test for NH_3_ synthesis over Co/SrNH at 300 °C, 0.9 MPa. Insets: Arrhenius plots over Co/SrNH at 0.9 MPa. **d** Catalytic activity of different Co catalysts in NH_3_ synthesis under 400 °C and 0.9 MPa. **e** Temperature dependence of the NH_3_ synthesis rates over Co/SrNH, Cs-Ru/MgO, wüstite-based Fe catalysts under 0.9 MPa. **f** Catalytic activity of TMs-supported SrNH (red) and SrO (blue) catalysts for NH_3_ synthesis at 0.1 MPa with Co- at 360 °C, Fe- and Ni- at 400 °C.

Subsequently, the catalytic performance of *Ae*NH for ammonia synthesis was investigated by loading Ni (Ni/*Ae*NH). The initial ammonia production rates of Ni/BaNH and Ni/SrNH are comparable (Fig. S2a), but much higher than that of Ni/CaNH catalyst. Meanwhile, BaNH support was not stable during the reaction and decomposed to unknown phases as indicated in XRD patterns (Fig. S2b–d), resulting an obvious degradation of catalytic activity (Fig. S2e). Thus, we focus SrNH as a model support in this work (Fig. S3). Figure 1a shows the catalytic activity of TM/SrNH as the function of temperature under 0.1 MPa. Among them, Co/SrNH shows the highest catalytic activity with the ammonia production rate of 5.0 mmol·g_cat._^−1^·h^−1^ at 340 °C (Table S1). The effluent NH_3_ concentration for Co/SrNH reached thermodynamic equilibrium above 360 °C and 0.1 MPa pressure. The calculated activation energies (E_a_) of the Co/SrNH and Fe/SrNH catalysts are ~50 kJ·mol^−1^ in the temperature range of 280–360 °C at 0.1 MPa, fairly smaller than Ni catalyst having E_a_ = 90 kJ·mol^−1^ (Fig. 1b). The higher E_a_ of Ni/SrNH is attributed to the different catalytic mechanism from Co/SrNH, Fe/SrNH and Ni/SrNH. The reaction orders with respect to N_2_, H_2_, and NH_3_ over TM/*Ae*NH catalysts are shown in Table 1 and Fig. S4. N_2_ reaction orders (α) for all tested catalysts are in the range of 0.8 ~ 1.2, whereas the H_2_ reaction orders (β) strongly depend on employed active metals. Compared to a bench mark catalyst Cs−Ru/MgO with negative H_2_ reaction orders, the positive value over TM/*Ae*NH catalysts indicate catalysts’ robustness against hydrogen poisoning. It should be noted that the H_2_ reaction order (β) of TM/*Ae*NH catalysts changed from 1.7 (Co/SrNH) and 1.6 (Fe/SrNH) to 0.2 (Ni/CaNH) and 0.1 (Ni/SrNH). Such a low H_2_ reaction order of Ni catalysts can be attributed to slow consumption of dissociated H^*^, resulting in a high coverage of adsorbed H^*^ on Ni surface. This is consistent with the relative larger E_a_ (ca. 90 kJ·mol^−1^) of Ni/CaNH (Fig. S5) and Ni/SrNH (Fig. 1b) compare to Co/SrNH and Fe/SrNH, which will be discussed later. Meanwhile, the large positive H_2_ reaction orders (β) of Co/SrNH and Fe/SrNH would be expected to lead a favorable pressure effect for ammonia synthesis, which can be confirmed by the linearly enhanced NH_3_ production rates over both Co/SrNH and Fe/SrNH catalysts under 0.9 MPa (Fig. S6). The Co/SrNH continuously produced ammonia at least for 100 h without clear degradation (Fig. 1c). High-angle annular dark-field scanning transmission electron microscopy (HAADF-STEM) images and corresponding Energy-dispersive X-ray spectroscopy (EDX) mapping results clearly demonstrated that Sr and N are uniformly dispersed on SrNH and the size of Co nanoparticle remained largely unchanged after long-term reaction (Figs. S7, S8), which indicates the stability of Co/SrNH. X-ray photoelectron spectroscopy (XPS) and Auger electron spectroscopy (AES) were further performed to check the surface element change of Co/SrNH before and after reactions. The results show that the valence state of Co and Sr species remains unchanged while the surface N content slightly decreased after the reaction (Fig. S9), which suggest that the lattice NH^2−^ may participate in the formation of NH_3_. The E_a_ of Co/SrNH under 0.9 MPa is largely unchanged with 0.1 MPa and much lower than that of conventional Ru based catalysts (133.0 kJ·mol^−1^, Fig. S10), suggesting that the catalytic mechanism is retained (Fig. 1c inset).

**Table 1:** Reaction orders of ammonia synthesis for various catalysts

Catalyst	N_2_ order (α)	H_2_ order (β)	NH_3_ order (γ)
Fe/SrNH	1.1	1.6	−1.6
Co/SrNH	1.2	1.7	−1.6
Ni/SrNH	1.2	0.1	−1.2
Ni/CaNH	1.2	0.2	−1.1
Cs-Ru/MgO	1.1	−0.5	−0.35

The ammonia production rate of Co/SrNH reaches 1686.7 mmol·g_Co_^−1^·h^−1^ at 400 °C and 0.9 MPa, which is much higher than those of reported Co- and Ni-based catalysts measured under a relative low space velocity (Fig. 1d, Table S2). It is noted that Co/SrNH with a low amount of Co loading (1.5 wt %) shows slightly lower reaction rates (Fig. S11a), but comparable TOFs to that of Co (4.8 wt %)/SrNH (Table S3), which should be ascribed to the smaller Co particle size as shown in Fig. S12. We are also aware that the apparent activation energy of Co/SrNH with a low amount of Co remains largely unchanged compared to Co (4.8 wt %)/SrNH (Fig. S11b), which indicated a similar reaction mechanism. The calculated turnover frequencies (TOFs) of Co (4.8 wt%)/SrNH is as high as 500 h^−1^, also far exceed those for previous reported Co based catalysts^13–15,22,32,35,39,40,47,51,53–56^ (Fig. S13, Table S3). Impressively, the activity of Co/SrNH even outperform the majority of reported Ru metal catalysts under similar reaction conditions^25,32,51,53,57–60^ (Fig. S13, Tables S4, S5). In terms of NH_3_ production rate, Co/SrNH outperforms the benchmark Cs-Ru/MgO catalyst and industrial wüstite-based Fe catalyst tested under the same reaction conditions (Fig. 1e). The specific activity of Co/SrNH (2.35 mmol·m^−2^·h^−1^, 400 °C, 0.9 MPa) is 5 times higher than those of our previously reported rare-earth metal nitride catalysts (Fig. S14). To unveil the high activity origin of Co/SrNH catalyst, we replaced SrNH support by SrO and test the ammonia synthesis under the same conditions. SrO was employed as the counter compound because they can be categorized to the related materials. If we consider NH^2–^ as an anion unit, both SrO and SrNH can be regarded as the rock salt type structure. Meanwhile, the two systems are distinct in terms of anion defect formation energy. In contrast to the relatively low NH^2–^ defect formation energy of SrNH (1.92 eV), the defect formation energy of O^2–^ of SrO reached 5.38 eV (Table S6), giving an ideal platform to investigate the effect of NH^2–^ defect to its catalytic mechanism. The NH_3_ production significantly decreased to near the detection limit over SrO supported Co and Fe catalysts, and even could not be detected for Ni/SrO, demonstrating that the NH^2–^ defect plays critical role during ammonia production in TM/SrNH (Fig. 1f).

**Fig. 2:**
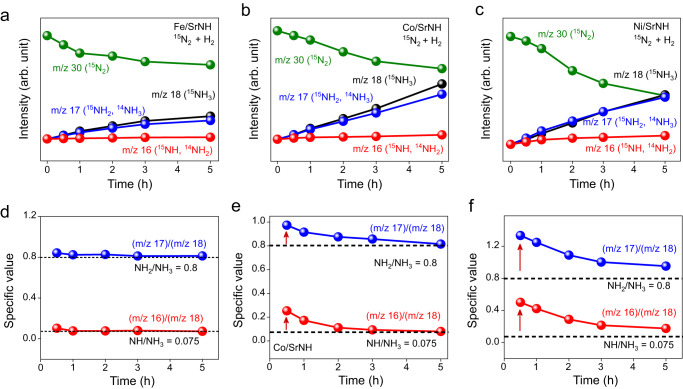
^15^N_2_/H_2_ isotopic experiments of TMs-SrNH catalysts. Reaction time profiles (**a**–**c**) for NH_3_ synthesis from ^15^N_2_ and H_2_, and (**d**–**f**) the ratio changes of *m/z* 17/18 and 16/18 over fresh Fe/SrNH, Co/SrNH, and Ni/SrNH catalysts.

To unveil the importance of the lattice NH^2−^ defects in the catalytic mechanism, the isotopic experiments were performed over TM/SrNH catalysts using ^15^N_2_/H_2_. With the increase of temperature, TM/SrNH continuously consumes ^15^N_2_ and lattice ^14^NH^2−^ of SrNH, producing ^15^NH_3_ and ^14^NH_3_. Therefore, the mass signals measured for m/z = 18 (^15^NH_3_), 17 (^15^NH_2_, ^14^NH_3_), and 16 (^15^NH, ^14^NH_2_) are gradually enhanced (Fig. 2a–c). The intensity ratios of *m/z* = 17/18 and 16/18 over Co/SrNH and Ni/SrNH catalysts were larger than the theoretical values (assuming all NH_3_ are derived from ^15^N_2_) of 0.8 (*m/z* 17/18) and 0.075 (*m/z* 16/18) at the initial state (at 0.5 h), suggesting that the formation of NH_3_ was derived from both ^15^NH_3_ and ^14^NH_3_ (Fig. 2b, c). Meanwhile, the *m/z* = 17/18 and 16/18 intensities for the Fe/SrNH catalyst are very close to the theoretical value at the initial state because the formation of ^14^NH_3_ is negligible (Fig. 2a). These results demonstrate that while the lattice ^14^NH^2−^ of Co/SrNH and Ni/SrNH are involved in the catalytic cycle, its participation is smaller over Fe/SrNH catalyst. Owing to much stronger Fe-N interaction, ^15^N_2_ and H_2_ are both activated on the Fe surface. These results show the multiple catalytic mechanism for TM/SrNH depending on TM.

**Fig. 3:**
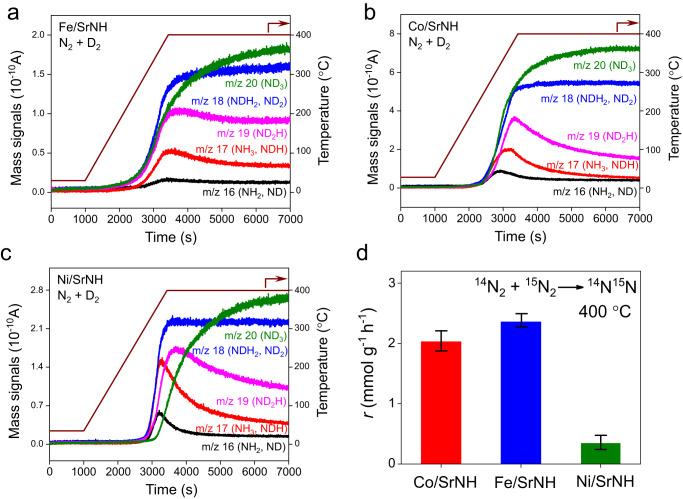
N_2_/D_2_ and ^14^N/^15^N isotopic experiments of TMs-SrNH catalysts. Surface reaction profiles for (**a**) Fe/SrNH, (**b**) Co/SrNH and (**c**) Ni/SrNH catalysts with the reaction gas of N_2_ and D_2_ at the temperature increased from room temperature to 400 °C. Prior to N_2_/D_2_ isotopic reaction, each sample is pretreated in H_2_ + N_2_ at 400 °C for 24 h. **d** Reaction rate of N_2_ isotope exchange over Fe/SrNH, Co/SrNH and Ni/SrNH catalysts at 26.7 kPa (^15^N_2_:^14^N_2_ = 1:4). Each sample is pretreated in H_2_ at 400 °C for 24 h before the ^14^N/^15^N isotopic experiment. Error bars represent the standard deviation from three independent measurements.

To furthermore investigate the catalytic mechanism, we subsequently conducted the isotope experiments employing N_2_ and D_2_. Ammonia (NH_3_) and its isotopic species (ND_3_, ND_2_H, NDH_2_, ND_2_, NDH, ND, and NH_2_) were detected with varying temperatures from 25 °C to 400 °C. It is noted that not only gas phase D_2_, but also lattice H species participate to ammonia production. In Fig. 3a, b, the ammonia isotopic fragments were detected simultaneously over Fe/SrNH and Co/SrNH above ca. 200 °C. It is in stark contrast to Ni/SrNH, in which the formation of ND_3_ was delayed compared to other fragments, i.e., ammonia isotopic fragments were detected above ca. 380 °C (Fig. 3c). Considering the weak interaction between Ni and N, Ni is unlikely responsible for N_2_ activation. Thus, such delayed *m/z* = 20 (ND_3_) signals may be attributed to the reaction of D* created on Ni with the lattice NH^2−^ and subsequent formation of NH^2−^ vacancy at SrNH support surface. The in situ generated NH^2−^ vacancy sites can serve as the activation centers for N_2_ and subsequent hydrogenation to NH_3_. In contrast, Co and Fe show much higher nitrogen affinity, giving rise to simultaneous activation of N_2_ and D_2_ on their surface and immediate formation of ND_3_. Accordingly, in ^14^N/^15^N isotopic exchange experiments (^14^N_2_ + ^15^N_2_ → ^14^N^15^N), the reaction rate of N_2_ isotope exchange over Co/SrNH (2.03 mmol·g^‒1^·h^‒1^) becomes 5 times higher than that for Ni/SrNH (0.35 mmol·g^‒1^·h^‒1^), and comparable to Fe/SrNH (2.36 mmol·g^‒1^·h^‒1^) (Fig. 3d). The apparent E_a_ for Ni/SrNH was obtained as 156.5 kJ mol^‒1^, showing the highest value among the three catalysts (Fig. S15). These results suggest that the N_2_ dissociation was preferred on Fe and Co metal, but unlikely on Ni metal, which is consistent with our hypothesis discussed above.

**Fig. 4:**
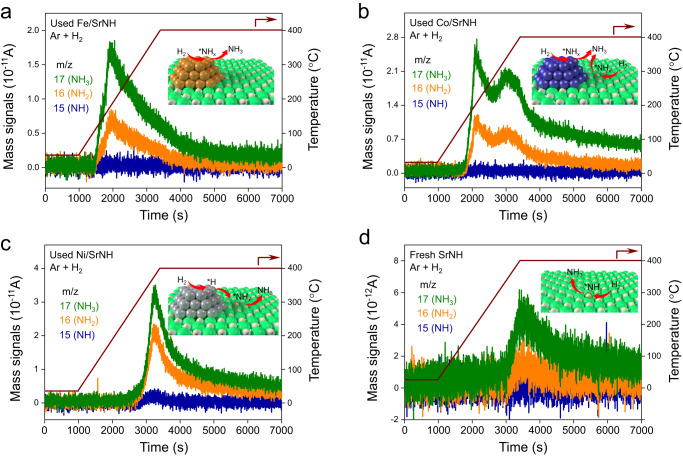
H_2_-Temperature programmed reaction (TPR) of TMs-SrNH catalysts. H_2_-TPR profiles for used (**a**) Fe/SrNH, (**b**) Co/SrNH, (**c**) Ni/SrNH, and fresh (**d**) SrNH catalysts under Ar and H_2_ (20 mL min^−1^ reaction gas, Ar/H_2_ = 1:1) at the temperature increased from room temperature to 400 °C. Prior to H_2_-TPR measurements, the samples denoted as used catalysts (**a**–**c**) were treated at 400 °C for 24 h under the NH_3_ synthesis condition (60 mL min^−1^ reaction gas, N_2_/H_2_ = 1:3).

H_2_-temperature programmed reaction (H_2_-TPR) measurements were further conducted to elucidate the reaction pathway of ammonia synthesis over TM/SrNH catalysts. Note that the dissociated N* and/or formed NH_*x*_ species remain on the surface of each used TM/SrNH catalyst. In used Fe/SrNH, the ammonia fragments (NH_3_, NH_2_ and NH) appeared at ca. 200 °C, consistent with the N_2_/D_2_ isotope experiments, suggesting that the Fe metal is mainly responsible for the N_2_ and H_2_ activation and subsequent NH_3_ formation (Fig. 4a). In it, SrNH support plays smaller contributions, consistent with the ^15^N/H_2_ isotopic results (Fig. 2a). Meanwhile, the desorption peak at ca. 200 °C was absent in Ni/SrNH due to the relatively weak nitrogen interaction of Ni metal (Fig. 4c), i.e., N_2_ is unlikely activated on Ni metal. Instead, a desorption peak at a higher temperature region (ca. 350 °C) can be identified for Ni/SrNH, which is attributed to the reaction between the dissociated H* and the lattice NH^2−^ in SrNH support (Fig. 4c). It should be emphasized that similar H_2_-TPR data is also confirmed for non-loaded SrNH (Fig. 4c, d), evincing that the peak at ca. 350 °C is indeed derived from the reaction between H* and the lattice NH^2−^ of SrNH. The greater amount of the desorbed ammonia in Ni/SrNH suggests that the enhanced NH_3_ formation is caused by the aid of dissociated H* from Ni. Different from Fe/SrNH and Ni/SrNH, Co/SrNH can be regarded as “hybrid” mechanism because of moderated interaction between Co and N. As shown in Fig. 4b, the Co/SrNH catalyst shows two major NH_3_ desorption peaks at ca. 200 °C and ca. 350 °C, which indicates the adsorption and activation of reactant N_2_ are associated with both loaded Co metal and NH^2−^ vacancy sites of SrNH support. It was thus demonstrated that the synergy of TMs and NH^2−^ vacancies plays a critical role for ammonia production, and multiple reaction pathways should be realized over Fe/SrNH, Co/SrNH and Ni/SrNH, respectively.

**Fig. 5:**
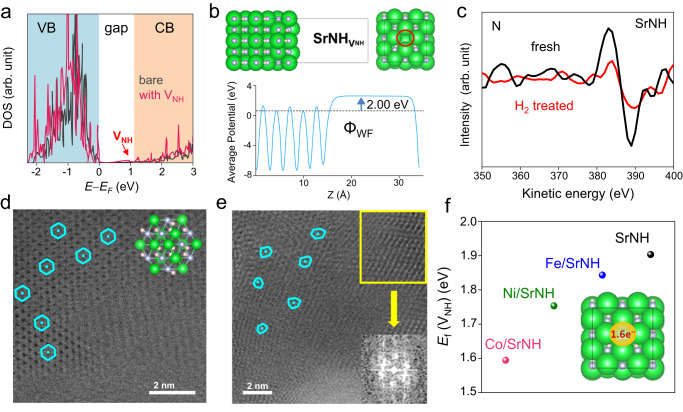
Calculation and characterizations of V_NH_ of TMs-SrNH catalysts. **a** Projected density of states (DOS) for SrNH with and without V_NH_. **b** Calculated work function of SrNH with surface NH^2−^ vacancy. **c** AES spectra for N, Sr and TMs of fresh and H_2_-treated SrNH. HRTEM image of (**d**) fresh and (**e**) H_2_-treated SrNH along the [111] direction. The inset of panel (**d**) shows the crystal structure of SrNH along the [111] direction. Sr, N and H atoms are represented as green, gray and light pink balls, respectively. The inset of (**e**) shows the corresponding FFT pattern of the yellow region. **f** V_NH_ formation energies E_V_ over bare SrNH and various TMs-SrNH catalysts. Inset shows the electron density in the region of V_NH_ site of bare SrNH.

To investigate the synergistic functionalities between TMs and NH^2−^ vacancies in TMs/SrNH catalysts, DFT calculations and detailed experimental characterizations were conducted. Bader charge analysis showed the three TMs are negatively charged, i.e., −0.08 (Fe), −0.14 (Co), and −0.10 (Ni) (Fig. S16). Co accepts more electrons than Fe because of its deeper 3d orbitals. Meanwhile, Ni accept fewer electrons than Co because its 3d orbitals are almost occupied. It is consistent with the XPS results, in which these 2*p* peaks of TMs shifted to lower binding energy region (Fig. S17). Such negatively charged TMs can be ascribed to the electron transfer from the NH^2^^−^ vacancy of SrNH to TMs (Fig. S18). Next, we evaluated how the NH^2−^ vacancy affects the electron donation ability of SrNH. Figure 5a shows the calculated density of states of SrNH with and without NH^2−^ vacancy. Compared with SrNH, an anionic electron state appeared between the valence and conduction band (Fig. S19), which is derived from the confined electron at the NH^2−^ vacancy of SrNH. Accordingly, the calculated work function (Ф_WF_) for NH^2−^ vacancy containing SrNH (SrNH_VNH_, Ф_WF_ ~ 2.0 eV, Fig. 5b) become smaller than that for defect-free SrNH (Ф_WF_ ~ 2.6 eV) (Fig. S20), showing that its electron donation ability is further strengthened by generating NH^2−^ vacancy. We thus considered that the confined anionic electrons can be donated effectively to TMs (Ф_WF_ ~ 5.0 eV) and thus facilitate N_2_ dissociation through the back-donation of electrons to anti-bonding π* orbitals of N_2_, and this electron transfer mechanism is particularly effective for Fe/SrNH and Co/SrNH.

To confirm the formation of the NH^2−^ vacancies, AES has been conducted to investigate the surface composition of fresh and H_2_-treated SrNH samples (Fig. 5c). The normalized intensity of N peak (ca. 387 eV) decreased clearly after the H_2_ treatment process, suggesting the reaction of surface NH^2−^ species transformed to NH_3_ by reacting with H*. The generation of surface NH^2−^ vacancies was further confirmed by high-resolution transmission electron microscopy (HR-TEM). Since the light element of N and H gave low contrast in HRTEM, the lattice Sr of SrNH support was detected with a hexagonal pattern along (111) direction (Fig. 5d). With the H_2_ treatment, a distortion of the hexagonal pattern of lattice Sr could be identified along the same direction (Fig. 5e), consistent with the formation of a substantial amount of surface NH^2−^ vacancies. The E_V_ are calculated as Co/SrNH (1.59 eV) < Ni/SrNH (1.75 eV) < Fe/SrNH (1.84 eV) < SrNH (1.92 eV) (Fig. 5f), indicating that the formation of NH^2−^ vacancies becomes easier upon TM-loading. Accordingly, the observed intensity change of N AES peak shows that the surface NH^2−^ was consumed by H_2_ and NH^2−^ vacancies are likely to be generated on SrNH surface (Fig. S21). To further strengthen our claim, we further performed X-ray photoelectron spectroscopy (XPS) characterizations to illustrate the presence of surface NH^2−^ deficiencies. As shown in Fig. S22, N 1*s* XPS peaks of fresh Co/SrNH are located at around 400 eV and the intensity of this N peak is significantly weakened after H_2_ treatment. After etching the surface by argon plasma, it is found that the N peak intensity was recovered and became the same level as that of fresh Co/SrNH. At the same time, the intensity of Sr XPS peaks is largely no changed. These results indicated a substantial amount of NH^2−^ vacancies are formed on SrNH surface after H_2_ treatment.

The NH^2–^ vacancies could be filled by other species such as H^−^ anions. To examine this, we also performed a H_2_-TPR and Ar-TPD experiment. In H_2_-TPR measurement, 0.1 g of fresh Co/SrNH was treated under pure H_2_ atmosphere at 400 °C for 24 h, and ~0.075 mmol NH_3_ could be detected (Fig. S23a). It means that 0.075 mmol lattice NH^2−^ was consumed during the H_2_ treatment. Subsequently, Ar-TPD measurements were conducted to estimate the incorporated H^−^ ions and the amount of the desorbed H_2_ was estimated to be 0.025 mmol (i.e., 0.05 mmol H^−^ ions, Fig. S23b), which indicates significant amount of NH^2−^ vacancies are occupied by H^−^ ions. DFT calculation also demonstrated that the NH^2−^ vacancy is favored to capture H^−^ as anions (Table S7). We acknowledge that H^−^ ions can be accommodated in partial NH^2−^ vacancy sites, which is also beneficial to the reduction of N_2_ to promote ammonia synthesis^37^. Meanwhile, it should be noted the desorbed H^−^ ions amount (0.05 mmol) was smaller than that of consumed lattice NH^2−^ (0.075 mmol), which demonstrate the existence of NH^2−^ vacancy in Co/SrNH. Such high amount of incorporated H^−^ ions would not lead to a change of the surface structure of SrNH (Fig. S24). XPS measurement shows that the valence states of the Sr species of H_2_-treated Co/SrNH are similar to that of fresh one (Fig. S22), suggesting the stable surface structure of SrNH during the H_2_ treatment. Most importantly, in Raman spectra, the absence of hydrogen vibration (broad bands at the wavelength of 400–1000 cm^−1^)^61,62^ further exclude the surface generation of SrH_2_ in H_2_-treated Co/SrNH (Fig. S25).

**Fig. 6:**
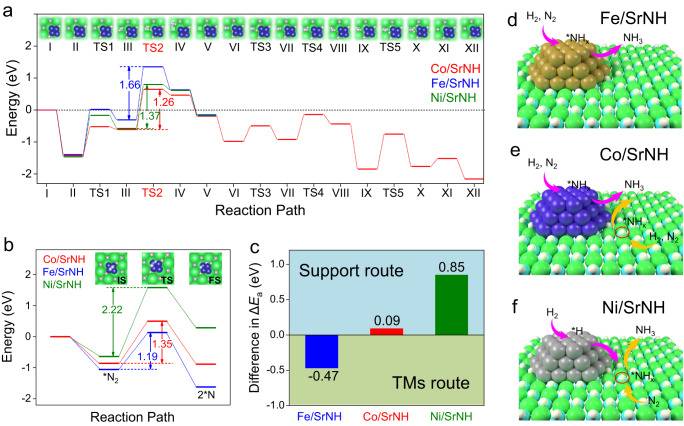
Theoretical calculation of TMs-SrNH catalysts. **a** Calculated energy profiles of support route for N_2_ activation and hydrogenation at the V_NH_ site of SrNH support (Support route) over Fe/SrNH, Co/SrNH and Ni/SrNH catalysts. Inset shows the structures of the intermediates and transition states (TSs) for the key elementary steps over Co/SrNH. **b** Calculated energy profiles of N_2_ activation on the surface of TMs (TMs route) in TMs-SrNH catalysts. **c** Calculated difference in energy barrier between the TMs and support reaction pathways for ammonia synthesis process on Fe-, Co- and Ni-loaded SrNH catalysts. ΔE_a_ is described as E_a_ (TM Route)−E_a_ (Support route). Proposed reaction pathway for ammonia synthesis over (**d**) Fe/SrNH, (**e**) Co/SrNH and (**f**) Ni/SrNH catalysts.

The energy profiles for lattice NH^2−^ hydrogenation over SrNH and N_2_ dissociation on TMs were investigated using DFT. As shown in Fig. 6a, the hydrogenation steps of the generated NH_2_^−^ species (TS2) gave the highest energy states among all the reaction coordination for each catalyst. It was detected that the energy barrier for NH_3_ formation on Fe/SrNH (1.66 eV) is much higher than those on Co/SrNH (1.26 eV) and Ni/SrNH (1.37 eV), reflecting the energetically unfavored pathway through the lattice NH^2−^ of the SrNH support over Fe catalyst (Tables S8–S9, Figs. S26–S28). Meanwhile, the comparable values over Co/SrNH and Ni/SrNH demonstrate that NH_3_ formation through the SrNH support are both energetically preferred. The slightly lower barrier of Co/SrNH indicates that the lattice NH^2−^ in Co/SrNH is more easily hydrogenated by H* than that in Ni/SrNH, which is consistent with the difference of calculated E_V_ (Fig. 5f).

Subsequently, the N_2_ dissociation on the surface of TMs were investigated. The E_a_s of TM/SrNH were calculated to be 1.19 eV (Fe), 1.35 eV (Co) and 2.22 eV (Ni), respectively (Fig. 6b, Fig. S29, Table S10). Here, we define the difference of the maximum energy barrier (ΔE_a_) between the TMs and SrNH support pathways as a rough descriptor of anticipated activity along each pathway (Fig. 6c), in which positive values of ΔE_a_ indicate that the SrNH support route is favored while negative values indicate TMs play more important role for ammonia synthesis. Fe/SrNH shows a ΔE_a_ value of −0.47 eV, suggesting that nitrogen and hydrogen reacted with each other majorly on Fe metal surface to produce NH_3_ (Fig. 6d). This is in good agreement with the ^15^N_2_/H_2_ isotopic experimental results, in which lattice NH^2−^ of SrNH support is not involved in ammonia product (Fig. 2a). In the case of Co/SrNH, the nearly zero value (0.09 eV) of ΔE_a_ implies the comparable energy barrier for both reaction pathways, i.e., both Co metal and SrNH support contributed to the formation of ammonia product, which can be confirmed by H_2_-TPR results as well (Fig. 4b). Remarkably, the comparable overall energy barrier of ~0.6 eV (ca. 57.9 kJ·mol^−1^) for SrNH support route (Fig. 6a) and ~0.5 eV (ca. 48.2 kJ·mol^−1^) for Co metal route (Fig. 6b) are also close to the experimentally obtained E_a_ from the Arrhenius plot (ca. 55 kJ·mol^−1^) (Fig. 1b). Therefore, we proposed a dual reaction pathway through the synergy of TMs and SrNH in Co/SrNH (Fig. 6e), in which the small work function (~2.0 eV) of SrNH support accounts for the strong electron donation ability that facilitates N_2_ activation on Co metal. Meanwhile, the formed NH^2−^ vacancy of the SrNH support provides additional active sites to the adsorption of N_2_ and then hydrogenation to NH_3_. As for Ni/SrNH catalyst, the weak interaction between Ni and N gave rise to the highest energy barrier for N_2_ dissociation as well as a positive value of ΔE_a_. Instead, N_2_ molecules are adsorbed and activated at the in situ formed NH^2−^ vacancy sites, and continuously react with H* from Ni, to realize a stable catalytic ammonia synthesis cycle over SrNH support (Fig. 6f), similar to the previously studied reported Ni/ReN system. The rate-determining step for the ammonia formation is associated with the combination of H* and NH^2−^ with a calculated energy barrier of 1.37 eV, and the overall energy barrier for NH_3_ formation through the hydrogenation process is roughly 0.8 eV (ca. 77.2 kJ·mol^−1^), comparable to the aforementioned E_a_ (ca. 90 kJ·mol^−1^) of Ni/SrNH (Figs. 1b and 6a). Such high energy barrier should hinder the diffusion of H* from Ni to SrNH in some degree, leading to a high H coverage over Ni catalysts, which accounts for almost zero H_2_ reaction order (β) (Table 1).

## Discussion

In our previously investigated ReN catalyst systems, the nitrogen vacancy not only provides active sites for N_2_ activation and hydrogenation to form NH_3_, but also reduces the work function of the ReN support, facilitating the N_2_ dissociation on loaded TMs metal. In this study, since the much larger anionic vacancy size of SrNH are realized compared to ReN, the energy barrier of lattice NH_x_ hydrogenation was significantly reduced over SrNH-based catalysts probably due to the steric effect (Fig. S30). Accordingly, the removal of lattice NH^2^^−^ from Ni/SrNH by H_2_ proceeds more easily than from Ni/CeN (Fig. S31). By employing NH^2^^−^ vacancy, the ammonia production over SrNH support became more efficient than that of ReN. On the other hand, a low work function is also a highlighted feature in SrNH with NH^2^^−^ vacancy, which promotes the activation of N_2_ molecules. Compared with the work function (Φ_WF_ = ~2.3 eV) of ReN_V_, a much lower work function (Φ_WF_ = ~2.0 eV) was realized by V_NH_ formation on the SrNH support (Fig. S32), enabling much stronger electron donation from SrNH to Co that can facilitate N_2_ cleavage on Co, which leads to significantly enhanced catalytic activity of Co metal for ammonia synthesis. Overall, an unprecedented high reaction rates were achieved on Co/SrNH as shown in Figs. S13, S14 and Table S1–S3. Therefore, the generated V_NH_ plays a dominant role during the reaction, and both Co and V_NH_ on SrNH served as the active centers for N_2_ activation and ammonia production.

The present work demonstrates that *Ae*NH can act as efficient supports for promoting various TMs catalysts in ammonia synthesis. The synergy of TMs and in situ formed NH^2−^ vacancy of *Ae*NH has a decisive effect on the reaction pathway and thus result in distinct catalytic performance. In Fe and Ni cases, the ammonia formation is separately realized at Fe metal and V_NH_ sites of the support, respectively. While, a combination of TMs and V_NH_ route can be achieved over Co/SrNH catalyst, which was proved to be the most efficient catalyst for ammonia synthesis among the different TMs/*Ae*NH catalysts investigated. The in situ generated V_NH_ of SrNH not only supplies surface active sites for N_2_ activation and hydrogenation to NH_3_, but also reduces the work function of SrNH support, promoting N_2_ dissociation as well as NH_3_ formation on Co metal. As a results, the catalytic activity of Co/SrNH far exceed the other reported Co- and Ni-based catalysts, and even higher than conventional Ru-based catalysts and industrial Fe-based catalyst. The present findings provide important information toward understanding the synergy effect of TMs and *Ae*NH support on the reaction pathway for ammonia synthesis.

## Methods

### Sample preparation

*Ae*H_2_ were prepared by the reaction of alkaline earth metal ingot (99.99% purity) with H_2_ gas by an Ar/H_2_ arc evaporation system^23^. In the arc evaporation process, the Ar and H_2_ partial pressures were set to 0.04 MPa and 0.01 MPa respectively, and the reaction current was set to 60 ~ 80 A. Subsequently, *Ae*NH were synthesized by reacting*Ae*H_2_ NPs under N_2_ atmosphere at 400 °C for 48 h. Iron carbonyl [Fe_2_(CO)_9_], cobalt carbonyl [Co_2_(CO)_8_] and nickelocene [Ni(C_5_H_5_)_2_] were used as TMs precursors respectively. Then each TMs precursor and *Ae*NH were mixed by hand-mill in agate mortar. The mixture was then heated in pure H_2_ flow to produce TMs-*Ae*NH. Since *Ae*NH is moisture sensitive, all of the preparation procedures were performed in the Ar-filled glovebox. The illustration of the preparation process and corresponding powder XRD patterns of the as prepared *Ae*H_2_ and *Ae*NH are shown in Fig. S33.

Other reference support materials, such as Ba-Ca(NH_2_)_2_, CeN, and C12A7:e^‒^ electride were prepared according to our previously reported method^26,32,47^, whereas MgO and SrO were commercially available products. Co-loading was conducted according to the same thermal reduction process as that used for SrNH. LaCoSi was fabricated by arc-melting process using stoichiometric amounts of lanthanum, cobalt, and silicon ingots. The obtained ingot was annealed at 1000 °C while wrapped in a sealed quartz tube for 5 days, and then purified by further annealing at 800 °C for 10 days. The preparation of Co_3_Mo_3_N was realized through a nitridation process by using CoMoO_4_ as precursor. CoMoO_4_ was heated in a quartz reactor under NH_3_ gas flow at 800 °C for 5 h. Before taking out the sample from the reactor, pure N_2_ was used to purge residual NH_3_ in the reactor. For the preparation of Cs-Ru/MgO, MgO was treated in high vacuum at 500 °C for 6 h and then mixed with Ru_3_(CO)_12_ in the Ar-filled glovebox. The obtained powder was sealed in a quartz tube and slowly heated to 250 °C for 2 h. The obtained dark gray powder was dispersed in an absolute ethanol solution of Cs_2_CO_3_ by stirring for 3 h, and then the solvent was removed by evaporation and the catalyst was dried in vacuum. The atomic ratio of Cs/Ru in the catalyst was 1.0 and the Ru content was determined to be 10.0 wt%.

### Catalytic reaction

Catalytic reactions were conducted in a fixed-bed flow system. In a typical run, 0.1 g catalyst was pretreated in a stream of N_2_:H_2_ = 1:3 under WHSV of 36,000 mL·g^−1^·h^−1^ and at 0.1 MPa using a temperature program of heating to 400 °C for 1 h and then holding at 400 °C for 2 h. The ammonia produced was monitored under steady-state conditions of temperature (250 ~ 400 °C) with a flow rate of 60 mL·min^‒1^ at 0.1 ~ 0.9 MPa. The ammonia produced was trapped in 5 mM sulfuric acid solution and the amount of NH_4_^+^ generated in the solution was determined using ion chromatography (Prominence, Shimadzu) with an electrical conductivity detector. Comparison of the catalyst performance was conducted under the same conditions.

Temperature-programmed reduction with H_2_ (H_2_-TPR) was also conducted in a fixed-bed flow system. 0.1 g catalyst was treated in a pure H_2_ flow (60 mL·min^−1^) at 0.1 MPa using a temperature program of heating (3 °C·min^−1^) to 400 °C and then holding at 400 °C for 24 h. The produced ammonia was dissolved in 5 mM sulfuric acid solution and the amount of NH^4+^ ions in the solution was identified using the same instrument for ammonia synthesis. Ar-Temperature-programmed desorption (Ar-TPD) (BELCAT-A, BEL) was also performed. Prior to measurements, 0.1 g catalyst was introduced into a quartz glass cell in an Ar-filled glovebox and the glass cell was heated (10 °C·min^−1^) in an Ar stream (50 mL·min^−1^), and the concentration of H_2_ was monitored with a thermal conductivity detector (TCD) and mass spectrometer (Bell Mass, BEL).

Since the active sites of Co/SrNH are consist of both transition metal and surface NH^2−^ vacancy, the calculation of turnover frequencies (TOFs) is based on the total amount of surface active sites including surface Co metal atoms and surface NH vacancy sites.

The amount of surface Co sites are derived from the average particle size observed by TEM. Assuming that the Co particles are semi-spherical, the TOF based on surface Co is calculated below:

The Co weight W_M_ [g] is calculated as Eq. (1):

1 $${W}_{{{\mbox{M}}}}=m\times \frac{{{\mbox{c}}}}{100}$$

where m [g] is the weight of the catalyst and c [%] is Co loading weight percentage.

The specific surface area of Co, A_M_ [m^2^ g^−1^], is calculated as Eq. (2):

2 $${{{{{{\rm{A}}}}}}}_{{{{{{\rm{M}}}}}}}=\frac{0.5\times 4{{\uppi }}{\left(\frac{{{{{{\rm{d}}}}}}}{2}\times {10}^{-9}\right)}^{2}\times {{{{{\rm{a}}}}}}}{{{{{{{\rm{W}}}}}}}_{{{{{{\rm{M}}}}}}}}$$

where d [nm] is the average diameter of the Co particles, a [count] is the number of Co particles, and W_M_ [g] is the weight of the Co.

The specific volume of Co, V_Co_ [m^3^ g^−1^], is calculated as Eq. (3):

3 $${{{{{{\rm{V}}}}}}}_{{{{{{\rm{M}}}}}}}=\frac{0.5\times \frac{4}{3}{{\uppi }}{\left(\frac{{{{{{\rm{d}}}}}}}{2}{\times 10}^{-9}\right)^{3}}\times \,{{{{{\rm{a}}}}}}}{{{{{{{\rm{W}}}}}}}_{{{{{{\rm{M}}}}}}}}=\frac{1}{{{{{{{\rm{\rho }}}}}}\times 10}^{6}}$$

where ρ [g cm^−3^] is the density of Co, which is 8.9 [g cm^−3^].

Based on Eqs. 2 and 3, A_M_ is solved as Eq. (4):

4 $${{{{{{\rm{A}}}}}}}_{{{{{{\rm{M}}}}}}}=\frac{6000}{{{{{{\rm{d}}}}}}\times {{{{{\rm{\rho }}}}}}}$$

The number of Co surface sites N_s_ [count] is calculated as Eq. (5):

5 $${{{\mbox{N}}}}_{{{\mbox{s}}}}=\frac{{{{\mbox{A}}}}_{{{\mbox{M}}}}\times {{{\mbox{W}}}}_{{{\mbox{M}}}}}{{{{\mbox{S}}}}_{{{\mbox{M}}}}\times {10}^{-18}}$$

where S_M_ [nm^2^] is the cross-sectional area per Co atom, which is 0.066 [nm^2^]. Here, since the average particle size of Co metal of Co (1.5 wt%)/SrNH and Co (4.8 wt%)/SrNH is 11 nm and 17 nm (Fig. S12), respectively, the estimated number of Ns is 1.4 × 10^18^ and 2.8 × 10^18^ for the Co (1.5 wt%) and Co (4.8 wt%).

The amount of surface vacancy sites was calculated from the concentration of top-layer NH_lattice_ of imide. From the unit cell of pure SrNH, the amount of top-layer NH_lattice_ was estimated as Eq. (6):

6 $${{{\mbox{N}}}}_{{{\mbox{NH}}}}=\frac{1}{{{{\mbox{a}}}}^{2}}\times {{{\mbox{S}}}}_{{{\mbox{BET}}}}\times {{{\mbox{g}}}}_{{{\mbox{cat}}}}$$

where a is the lattice parameter of SrNH (a = 5.64 ×10^−10 ^m), S_BET_ is the surface area of Co/SrNH (S_BET_ = 15.9 m^2^·g^−1^), and g_cat_ (=0.1 g) is the amount of catalyst used during the experiment. According to the lattice structure, one NH site presents per the area of 3.18 × 10^−19^ m^2^. Therefore n_NH_ ~ 5.03 × 10^18^ of NH sites exist in the used sample according to the lattice structure of SrNH. According to our experimental results in Figure S23, around 2/3 of the NH defects are occupied by H^−^, which indicates that 1/3 of the surface NH vacancies can serve as the catalytic active sites. Thus, the amount of surface vacancy active sites should be 1.7 × 10^18^.

According to above calculation of the amount of surface active sites, the TOF [h^−1^] is calculated as Eq. 7:

7 $${{\mbox{TOF}}}=\frac{{{{\mbox{r}}}}_{{{{\mbox{NH}}}}_{3}}}{{{{\mbox{N}}}}_{{{\mbox{s}}}}{{\mbox{+}}}{{{\mbox{N}}}}_{{{\mbox{NH}}}}}\times {10}^{-6}\times 6.02\times {10}^{23}$$

where ${r}_{{{{\mbox{NH}}}}_{3}}$[μmol g^−1^ h^−1^] is the ammonia synthesis rate.

### Kinetic analysis

The apparent *E*_a_ were calculated from Arrhenius plots for the reaction rates, which were <20% of that at equilibrium. Measurement of the reaction orders for N_2_ and H_2_ was conducted with Ar gas as a diluent to ensure a total flow of 60 mL min^−1^ when changing the flow rate of N_2_ and H_2_. The reaction orders were estimated by using the following Eqs:

8 $${{{{{{\rm{r}}}}}}={{{{{\rm{k}}}}}}\times {{{{{\rm{P}}}}}}}_{{{{{{{\rm{N}}}}}}}_{2}}^{{{{{{\rm{\alpha }}}}}}}{\times {{{{{\rm{P}}}}}}}_{{{{{{{\rm{H}}}}}}}_{2}}^{{{{{{\rm{\beta }}}}}}}{\times {{{{{\rm{P}}}}}}}_{{{{{{{\rm{NH}}}}}}}_{3}}^{{{{{{\rm{\gamma }}}}}}}$$

9 $${{\mbox{r}}}=\frac{1}{{{\mbox{W}}}}\frac{{{\mbox{d}}}{{{\mbox{y}}}}_{0}}{{{\mbox{d}}}\frac{1}{{{\mbox{q}}}}}$$

10 $$\log {y}_{0}=\log {\left(\frac{{{\mbox{C}}}}{q}\right)}^{\frac{1}{m}}$$

11 $$r=\frac{1}{W}\times \frac{{{\mbox{C}}}}{m}\times {{y}_{0}}^{1-m}$$

12 $${{{{{{\rm{C}}}}}}={{{{{\rm{k}}}}}}}_{2}{\times {{{{{\rm{P}}}}}}}_{{{{{{{\rm{N}}}}}}}_{2}}^{{{{{{\rm{\alpha }}}}}}}{\times {{{{{\rm{P}}}}}}}_{{{{{{{\rm{H}}}}}}}_{2}}^{{{{{{\rm{\beta }}}}}}}$$

where *r*, *W*, *y*_0_, *q*, and (1-*m*) represent the reaction rate of the ammonia synthesis, the catalyst weight, the mole fraction of NH_3_ at the reactor outlet, the flow rate, and the reaction order with respect to NH_3_ (*γ*). Finally, the *α* and *β* can be determined by plotting the logarithm of “*C*” vs that of N_2_ or H_2_ partial pressure.

### Isotopic experiments

Ammonia synthesis was performed in a U-shaped glass reactor connected to a closed gas circulation system. The reactants, ^15^N_2_ (98%) and H_2_ with a ratio of 1:3, were introduced into the system with a total pressure of 60 kPa and then heated to 400 °C. The composition of the circulating gas through the system was detected by utilizing a quadrupole mass spectrometer (M-101QA-TDM, Canon Anelva Corp.), using Ar as the carrier gas. To overcome the limitations of reactant gas diffusion and adsorption/desorption, a circulating pump was introduced into the system. Masses with m/z values of 2, 16, 17, 18, 28, 29, and 30 were monitored over time to track the progress of the reaction.

The experiment of N_2_/D_2_ and H_2_-Temperature-programmed reaction (H_2_-TPR) was carried out in a fixed-bed flow system. Both N_2_/D_2_ and Ar/H_2_ gases at a flow ratio of 1 were used with a total flow rate of 20 mL·min^−1^. The fixed-bed reactor was then heated to 400 °C at a rate of 10 °C/min and 0.1 MPa. To analyze the reaction products, an online mass spectrometer (ANELVA, Quadrupole Mass Spectrometer) was used. The masses *m/z* = 16, 17, 18, 19, and 20 were monitored over time to track the progress of the reaction.

The N_2_ isotope exchange experiment was conducted in a closed gas circulation system equipped with a U-shaped glass reactor. The reactants, a mixture of ^15^N_2_ and ^14^N_2_ in a 1:4 ratio, were introduced into the system at a total pressure of 20 kPa. Afterward, the mixture was heated to 400 °C until reaching adsorption equilibrium. The gas in circulation was continuously monitored using a quadrupole mass spectrometer (M-101QA-TDM, Canon Anelva Corp.), measuring the mass-to-charge ratios (*m/z*) of 28, 29, and 30 as a function of time.

### Sample characterization

The crystal structure was analyzed using XRD (D8 Advance, Bruker) with Cu Kα radiation (*λ* = 0.15418 nm). The sample was put in an X-ray transmitting capsule to protect it from oxidation. High-resolution transmission electron microscopy (HR-TEM) images were obtained using a JEOL JEM-ARM300F atomic resolution analytical electron microscope operated at an accelerating voltage of 300 kV. X-ray photoelectron spectroscopy (XPS; ESCA-3200; Shimadzu) measurements were performed using Mg Kα radiation at <10^−6^ Pa (8 kV bias voltage applied to the X-ray source). XPS data were corrected according to the C (carbon) 1 s peak (binding energy = 284.6 eV). Auger electron spectra were obtained with 10 keV primary electrons using a scanning Auger nanoprobe system (PHI 710, Ulvac-Phi). Raman spectra were measured with a spectrometer (HR-800, Horiba Jobin Yvon), using a laser with a wavelength of 457 nm. Nitrogen sorption measurements (BELSORP-mini II, BEL, Japan) were applied to evaluate the Brunauer-Emmett-Teller surface areas of the catalysts.

### Theoretical calculations

All DFT calculations were got through the Vienna ab initio simulation package (VASP)^63^. The management of the electron exchange and correlation energy was generalized gradient approximation method with the Perdew–Burke–Ernzerhof (PBE) exchange–correlation functional^64^, while the projector augmented wave (PAW) method^65,66^ was employed to describe the core electrons. The description of valence electrons was using plane wave basis kinetic energy cut-off value with 450 eV. A k-point mesh of 2 × 2 × 1 was used from the Gamma center. Meanwhile, in the case of system, the unit cell was 16.82 × 16.82 × 39 Å^3^, the thickness of the vacuum layer was 25 Å. The upper two layers of each slab were allowed to relax (Fig. S34), while the bottom layers were constrained to their original positions. The 6-atoms metal cluster with octahedron structure was loaded on slab before relaxation. All models were fully optimized until the energy and forces are converged to 1 × 10^−5^ eV and 0.0257 eV Å^−1^, respectively. The *Ae*NH (001), based on the surface energy of the three facets with low miller index, was selected for study because it was the most stable facet (Table S11). To introduce TM cluster, we chose six Co atoms model with an octahedron structure since 1-4-1 is the most stable 3D configuration^67^. Then, two sites were considered for the cluster location. Table S12 shows the total energy of Co cluster loaded on NH site and Sr site of SrNH (001) facet respectively. It is clear that NH site loading is more energy favored. For TS calculation, the parameters of CI-NEB^68^ were kept the same as the structure relaxation. Three transition states were interpolated linearly between initial state and final state by VASPKIT package^69^.

The formation energy of NH vacancy was conducted following Eqs. (13) and (14):

13 $$\Delta {{{{{{\rm{E}}}}}}}_{{{{{{\rm{V}}}}}}}={{{{{{\rm{E}}}}}}}_{{{{{{\rm{V}}}}}}}+{{{{{{\rm{E}}}}}}}_{{{{{{\rm{NH}}}}}}}-{{{{{{\rm{E}}}}}}}_{{{{{{\rm{slab}}}}}}}$$

14 $${{{{{\rm{}}}}}}{{{{{{\rm{E}}}}}}}_{{{{{{\rm{NH}}}}}}}=({{{{{{\rm{E}}}}}}}_{{{{{{\rm{nitrogen}}}}}}}+{{{{{{\rm{E}}}}}}}_{{{{{{\rm{hydrogen}}}}}}})/2$$

Meanwhile, the formation energy of NH vacancy with the help of H was also conducted by E_NH_ = E_NH3_ – E_H2_. And the tendency of the vacancy formation energy with respect to NH_3_ – H_2_ is almost the same to those of N_2_ and H_2_ (Table S13).

All the binding energies of intermediates were E = E_X_ − E_slab_ – E_*X_ based on following Eq. (15):

15 $${{{{{\rm{X}}}}}}+{{{{{\rm{slab}}}}}}\to*{{{{{\rm{X}}}}}}$$

while X was the intermediates during ammonia synthesis, and *X was the intermediates adsorbed on catalyst.

### Supplementary information

Supplementary Information

Peer Review File

## Data Availability

The data generated in this study are presented in the main text and Supplementary Information, and can be obtained from the corresponding authors upon reasonable request.
