# Supplementary material for: Multiple reaction pathway on alkaline earth imide supported catalysts for efficient ammonia synthesis
Source: Nat Commun. 2023 Oct 11;14:6373. doi: 10.1038/s41467-023-42050-7 (PMC10567757; doi:10.1038/s41467-023-42050-7)
Supplement: Supplementary file 1 — Supplementary Information [file 41467_2023_42050_MOESM1_ESM.pdf]

Supplementary Information

Multiple reaction pathway on alkaline earth imide supported catalysts for efficient ammonia synthesis

Zichuang Li[1], Yangfan Lu[2], Jiang Li[3], Miao Xu[4], Yanpeng Qi[5,6,7], Sang-Won Park[3], Masaaki Kitano[3]*, Hideo Hosono[3]*, Jie-Sheng Chen[1] and Tian-Nan Ye[1]*

[1]Frontiers Science Center for Transformative Molecules, School of Chemistry and Chemical Engineering, Shanghai Jiao Tong University, Shanghai 200240, China.

[2]College of Materials Science and Engineering, National Engineering Research Center for Magnesium Alloys, Chongqing University, Chongqing 400044, China.

[3]Materials Research Center for Element Strategy, Tokyo Institute of Technology, 4259 Nagatsuta, Midori-ku, Yokohama 226-8503, Japan.

[4]State Key Laboratory of Space Power Sources, Shanghai Institute of Space Power-Sources, Shanghai 200245, China.

[5]School of Physical Science and Technology, Shanghai Tech University, Shanghai 201210, China.

[6]ShanghaiTech Laboratory for Topological Physics, Shanghai Tech University, Shanghai 201210, China.

[7]Shanghai Key Laboratory of High-resolution Electron Microscopy, Shanghai Tech University, Shanghai 201210, China.

*e-mail: kitano.m.aa@m.titech.ac.jp, hosono@mces.titech.ac.jp, ytn2011@sjtu.edu.cn

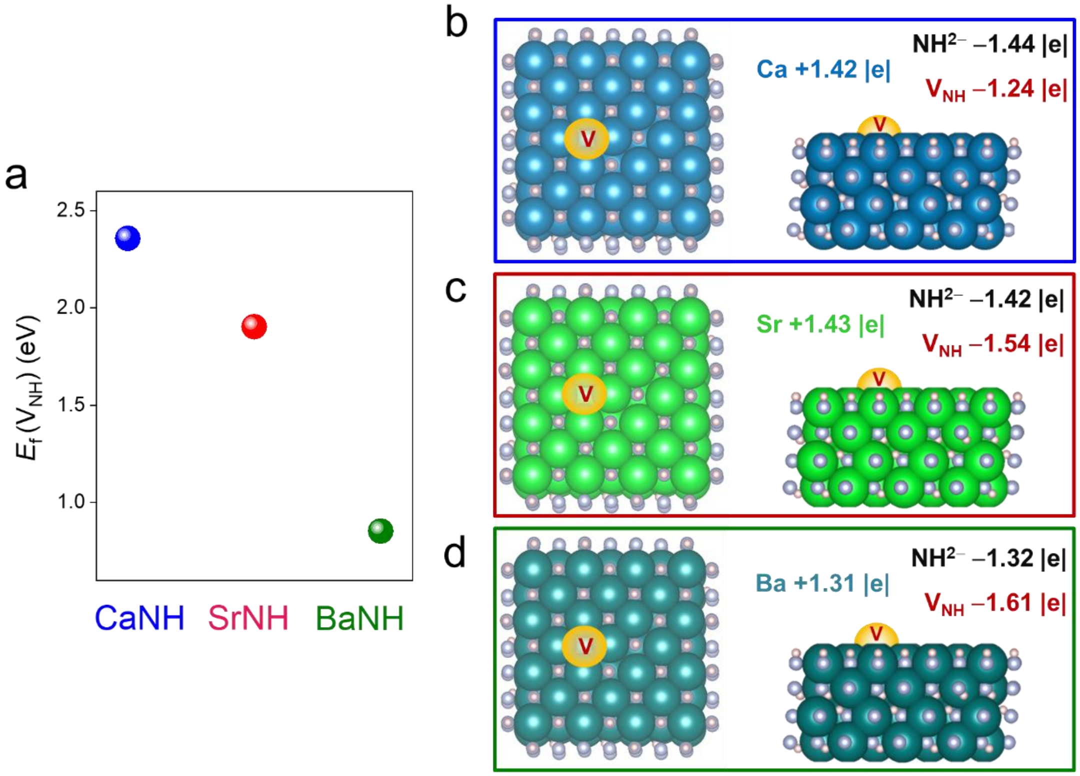

Figure S1 DFT calculations of NH^{2-} vacancy formation over *Ae*NH (*Ae* = Ca, Sr, Ba). (a) NH^{2-} vacancy formation energies [$E_f(V_{NH})$] of bare CaNH, SrNH and BaNH; (b-d) Electron density in the region of NH^{2-} vacancy site and corresponding calculated Bader Charges.

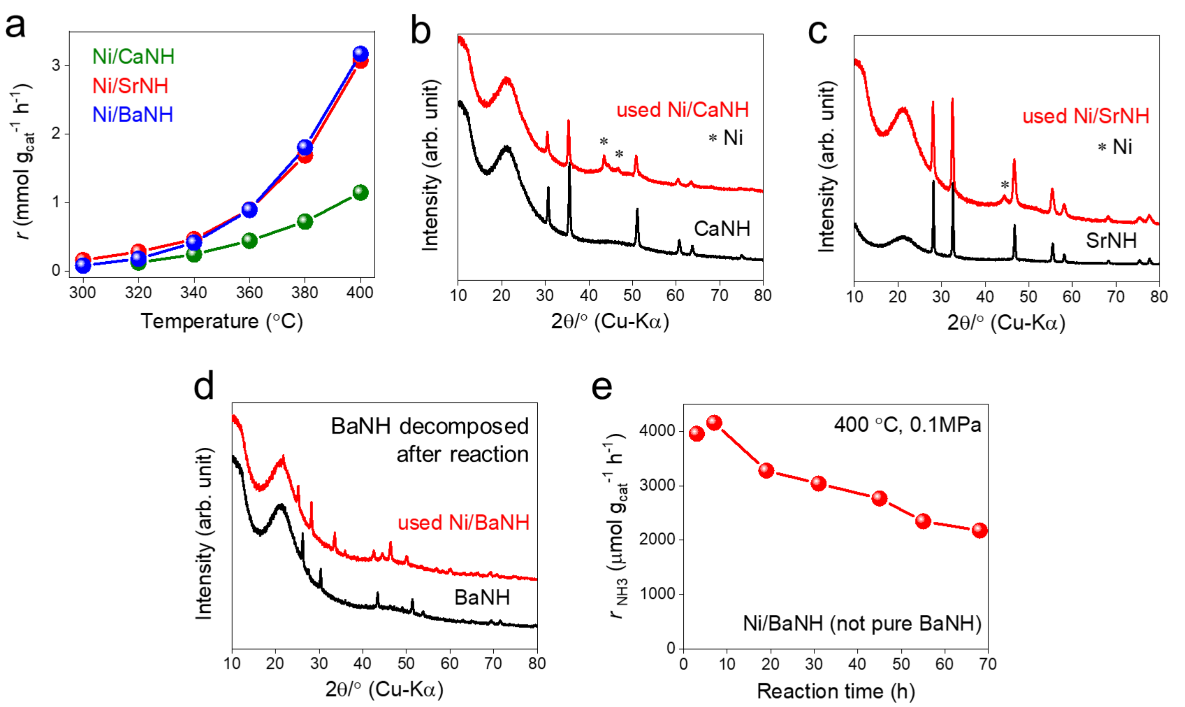

Figure S2 Catalytic performance and crystal structure of *Ae*NH and Ni/*Ae*NH (*Ae* = Ca, Sr, Ba). (a) Temperature dependence of the NH_3 synthesis activity of Ni loaded CaNH, SrNH and BaNH catalysts at 0.1 MPa. Powder XRD patterns for Ni loaded (b) CaNH, (c) SrNH and (d) BaNH catalysts after ammonia synthesis. (e) Time courses for ammonia synthesis over Ni/SrNH. (Reaction conditions: catalyst, 0.1 g; WHSV, 36,000 mL·g_{cat}^{-1}·h^{-1}).

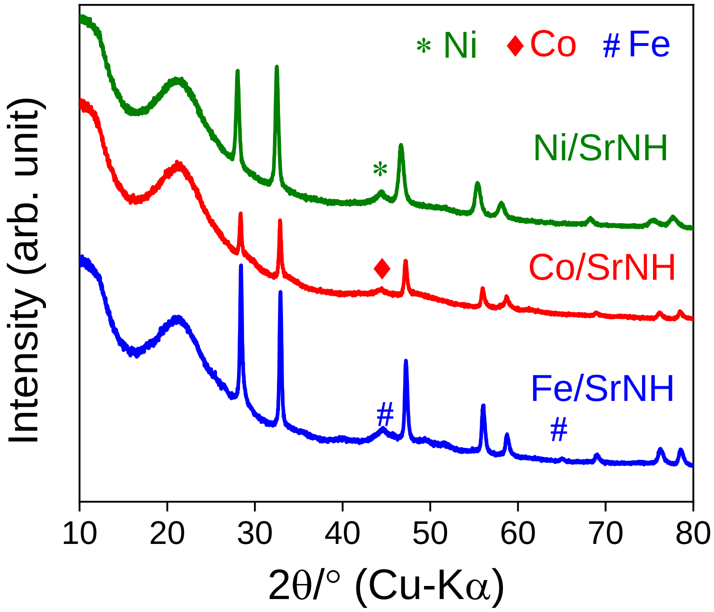

Figure S3 Crystal structure of *TMs*/SrNH (*TMs* = Fe, Co, Ni). Powder XRD patterns for Fe/SrNH, Co/SrNH and Ni/SrNH catalysts after ammonia synthesis.

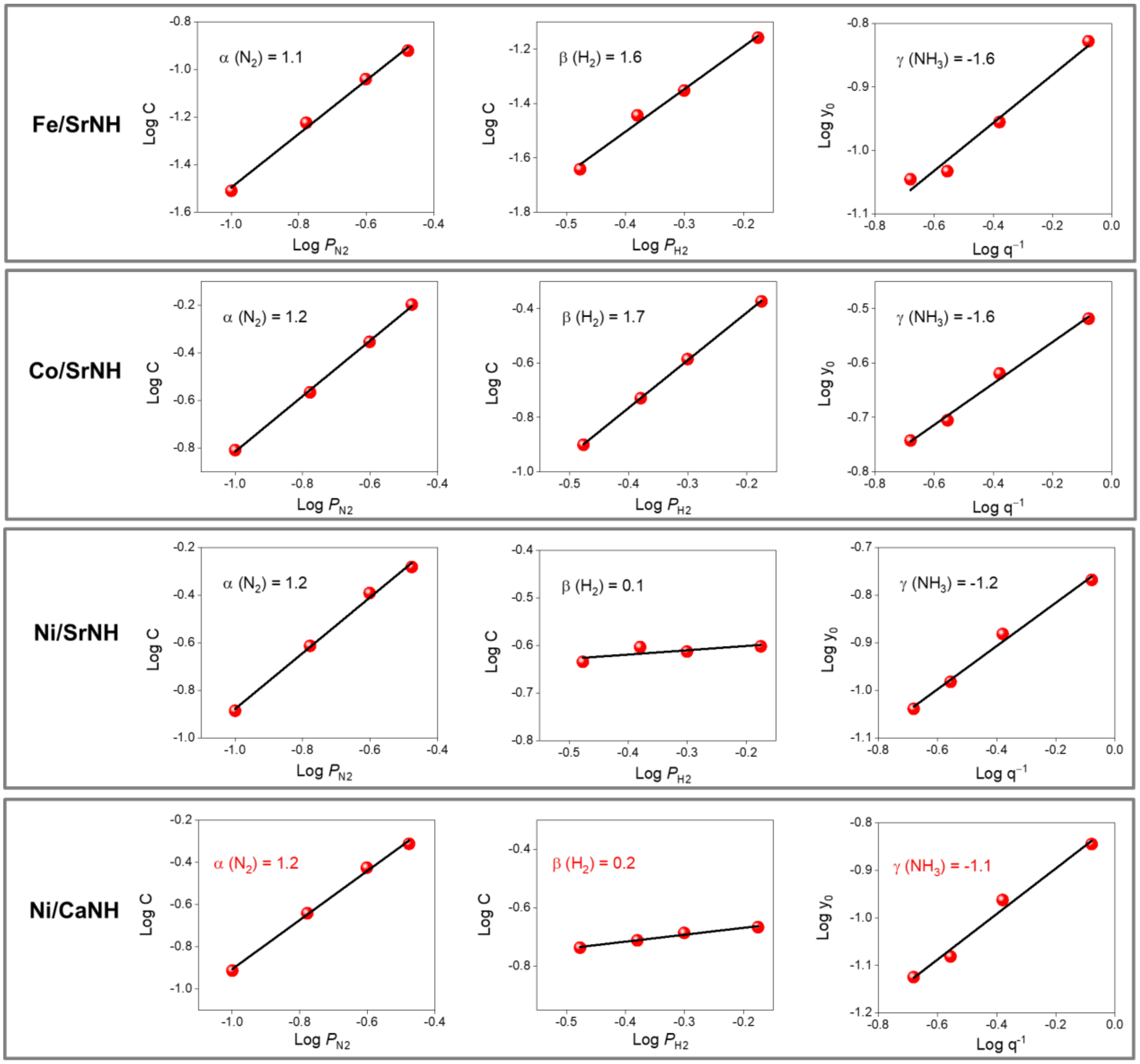

Figure S4 Kinetic reaction orders of *TMs*/SrNH (*TMs* = Fe, Co, Ni) and Ni/CaNH. Dependence of reaction rate (C) on the partial pressure of N_2 and H_2 on Fe/SrNH, Co/SrNH, Ni/SrNH and Ni/CaNH catalysts. Relation between effluent NH_3 mole fraction (y0) and total flow rate (q).

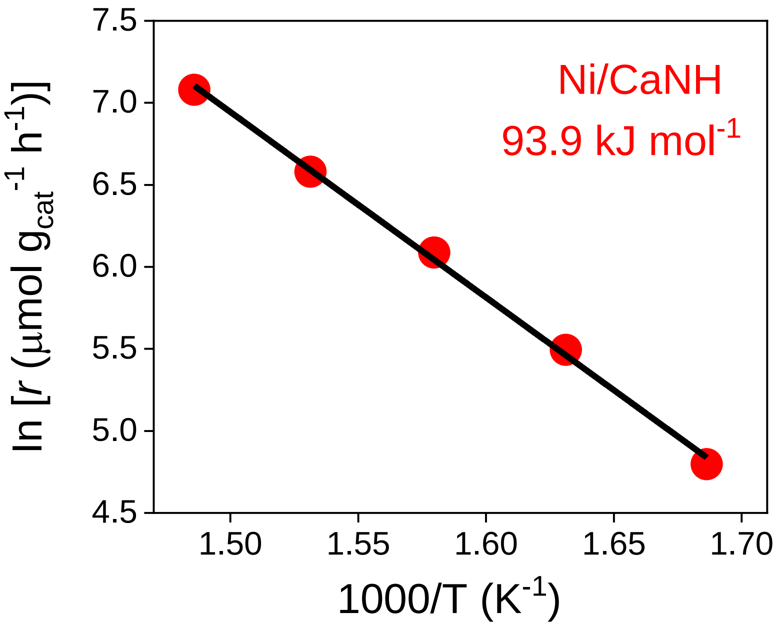

Figure S5 Apparent activation energies (E_a) of Ni/CaNH. Arrhenius plots for ammonia synthesis over Ni/CaNH. Reaction conditions: catalyst, 0.1 g; WHSV, 36,000 mL·g_{cat}^{-1}·h^{-1}, 0.1 MPa.

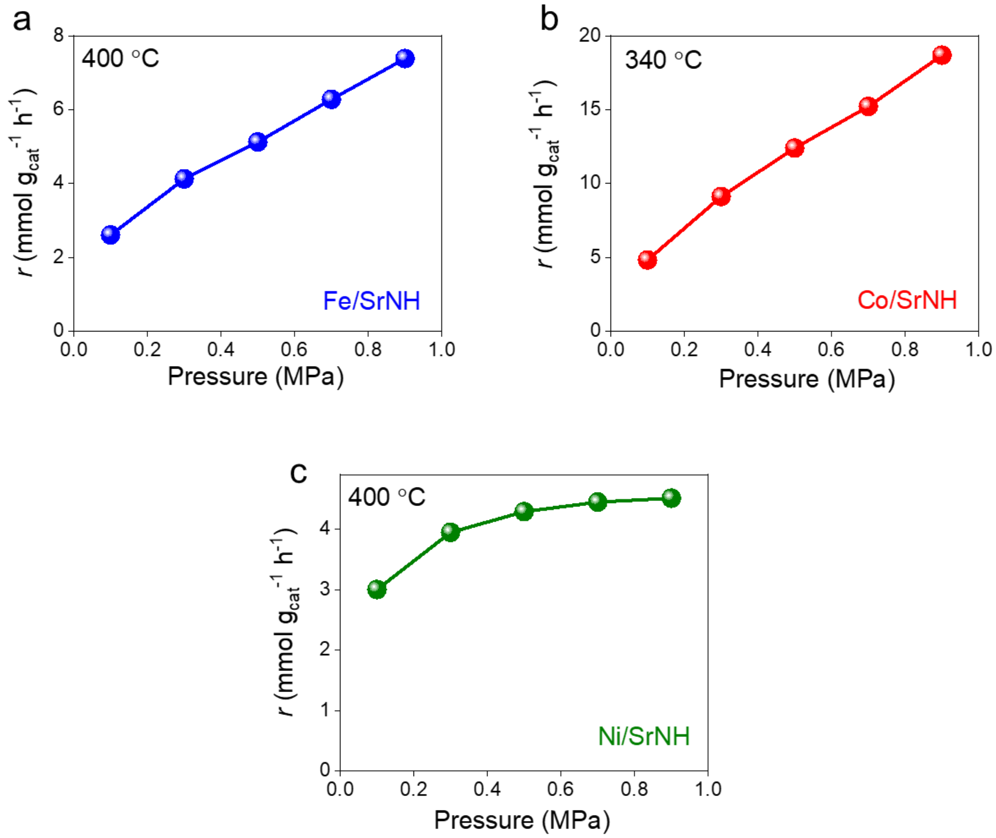

Figure S6 Pressure effect over *TMs*/SrNH (*TMs* = Fe, Co, Ni). Pressure dependence of the ammonia synthesis activity over (a) Fe/SrNH, (b) Co/SrNH and (c) Ni/SrNH. (Reaction conditions: catalyst, 0.1 g; WHSV, 36,000 mL·gcat^{-1}·h^{-1}).

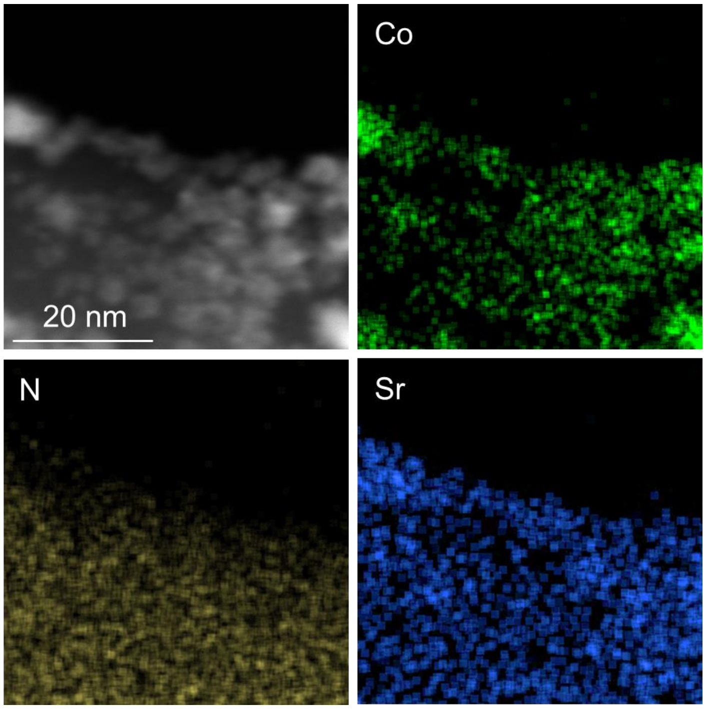

Figure S7 Morphology characterization of fresh Co/SrNH. HAADF-STEM image and EDX mapping results for Co, N, and Sr in fresh Co/SrNH catalyst.

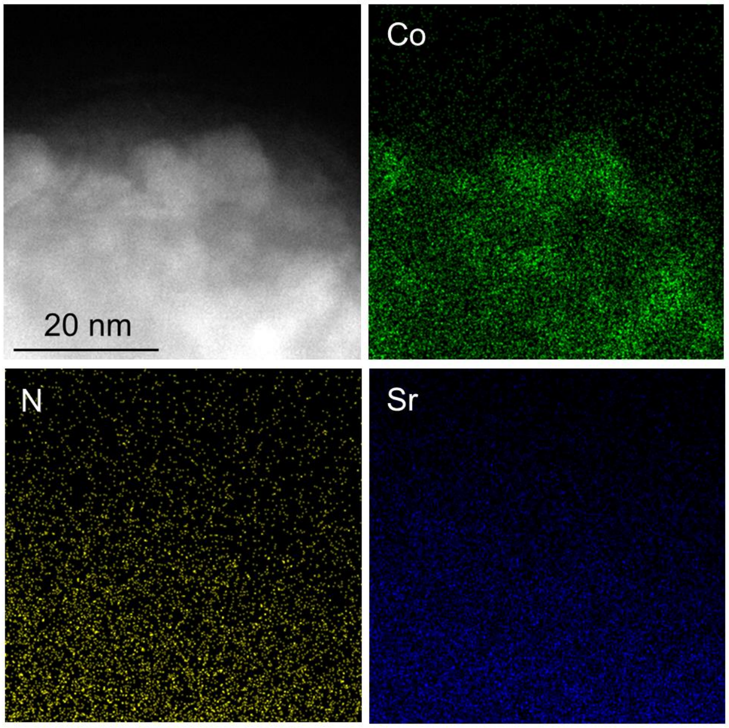

Figure S8 Morphology characterization of used Co/SrNH. HAADF-STEM image and EDX mapping results for Co, N, and Sr in used Co/SrNH catalyst.

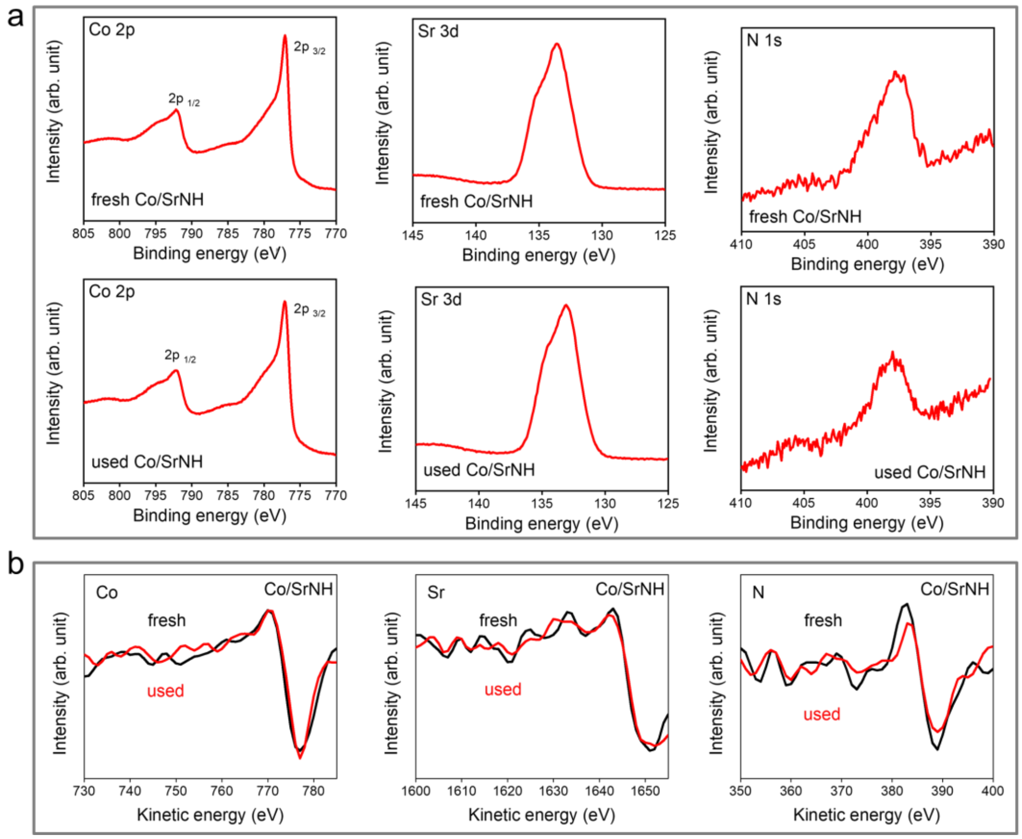

Figure S9 Surface characterization of Co/SrNH. (a) XPS and (b) AES spectra of Co2p, Sr 3d and N 1s in fresh and used Co/SrNH catalysts.

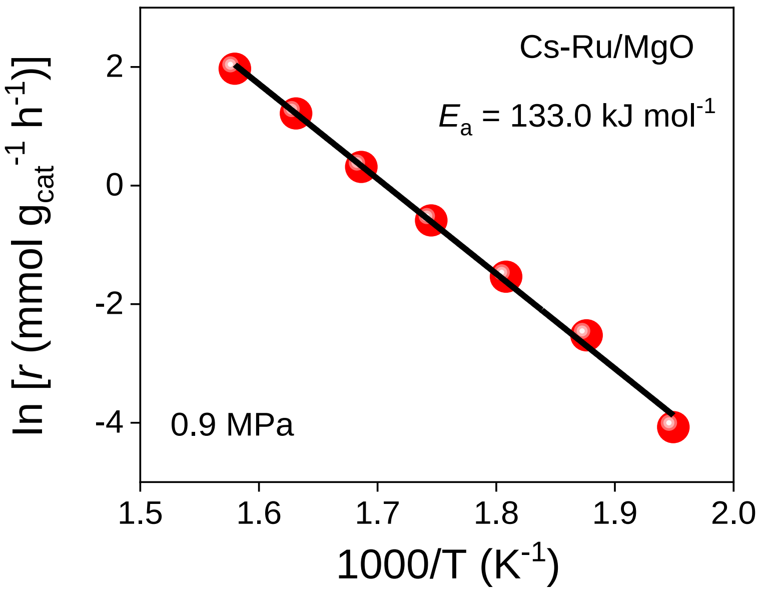

Figure S10 Apparent activation energies (E_a) of Cs-Ru/MgO. Arrhenius plots for ammonia synthesis over Cs-Ru/MgO. Reaction conditions: catalyst, 0.1 g; WHSV, 36,000 mL·g_{cat}^{-1}·h^{-1}, 0.9 MPa.

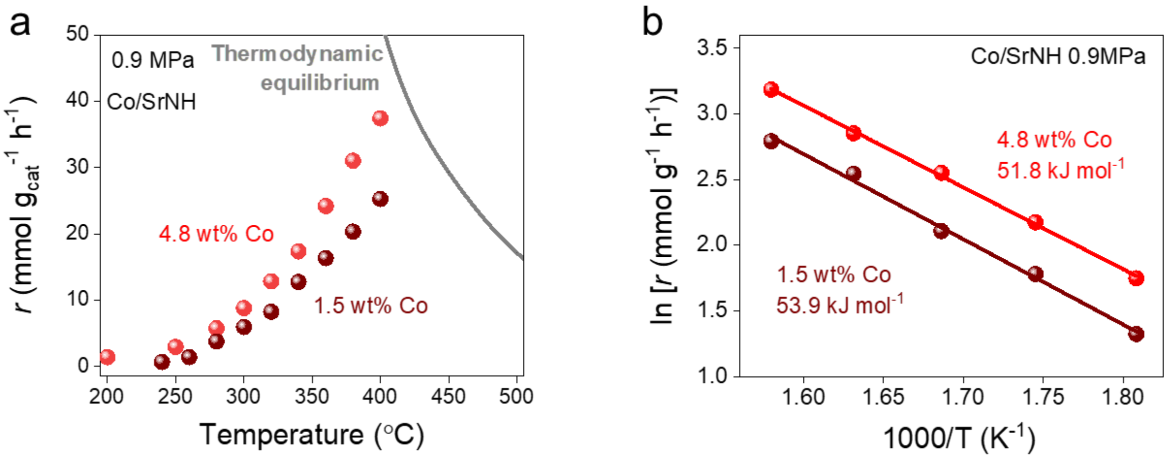

Figure S11 Catalytic performance and apparent activation energies (E_a) of Co/SrNH. (a) Temperature dependence of the ammonia synthesis rates and (b) Arrhenius plots for ammonia synthesis over Co/SrNH with 4.8 and 1.5 wt% Co loading amount under 0.9 MPa.

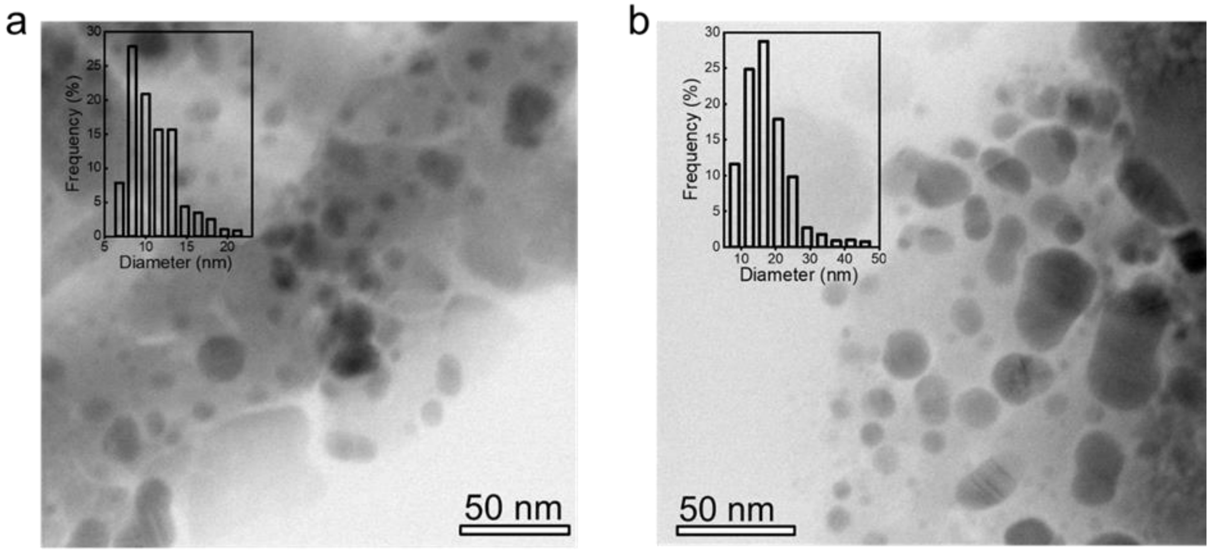

Figure S12 Morphology and particle size analysis of Co/SrNH. HRTEM image and corresponding particle size distribution of Co metal over Co/SrNH with (a) 1.5 and (b) 4.8 wt% Co loading amount after ammonia synthesis reactions under 0.9 MPa.

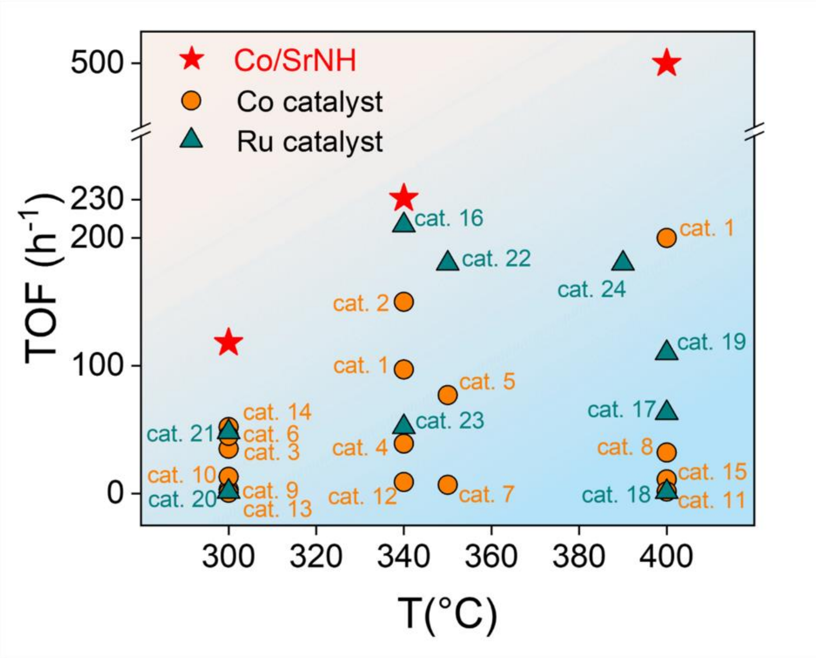

Figure S13 Activity comparison of Co/SrNH and reported Co and Ru catalysts under similar reaction conditions. All TOF values are determined by ammonia synthesis rate divided by the number of surface active sites.

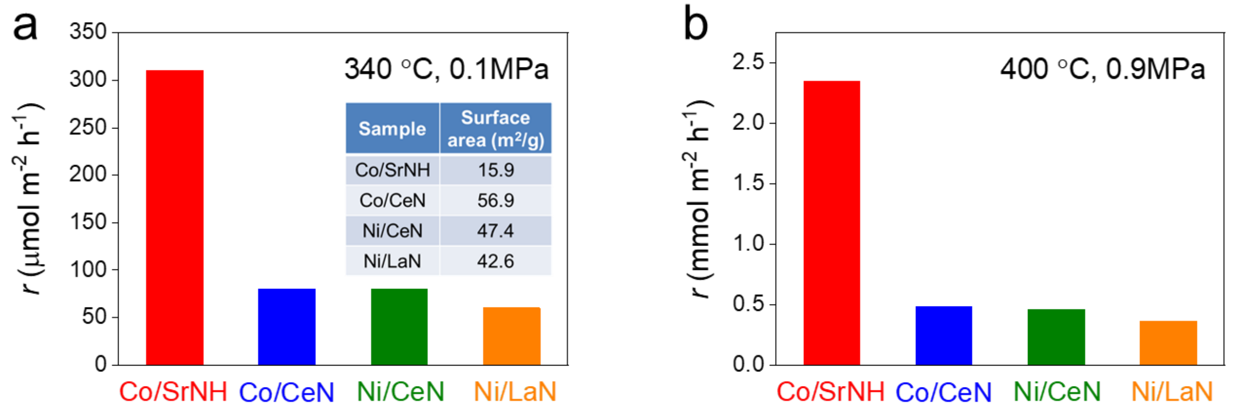

Figure S14 Activity comparison of Co/SrNH and reported CeN based catalysts. Specific activity for ammonia synthesis over Co/SrNH, Co/CeN, Ni/CeN and Ni/CeN catalysts at (a) 340 °C, 0.1 MPa and (b) 400 °C, 0.9 MPa. Reaction conditions: catalyst, 0.1 g; WHSV, 36,000 mL·g_{cat}^{-1}·h^{-1}.

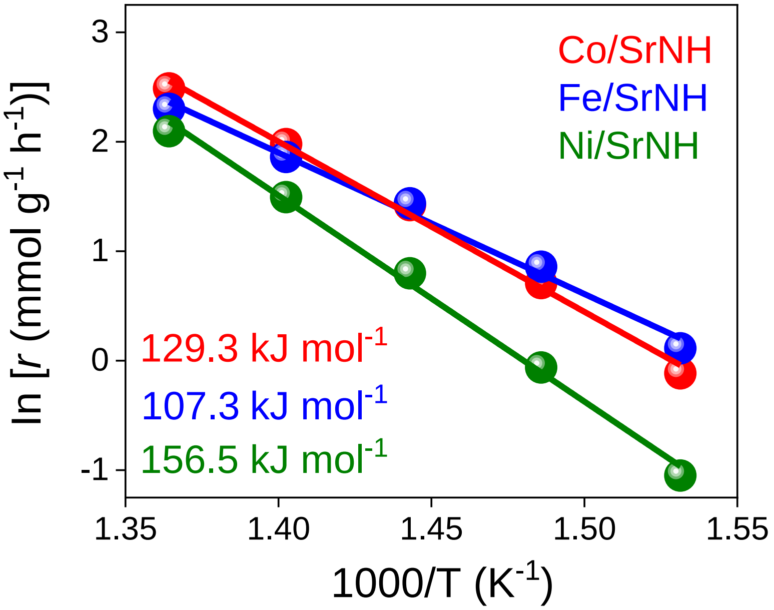

Figure S15 Apparent activation energies (E_a) of *TMs*/SrNH (*TMs* = Fe, Co, Ni). Arrhenius plots for N_2 isotope exchange over Fe/SrNH, Co/SrNH and Ni/SrNH catalysts.

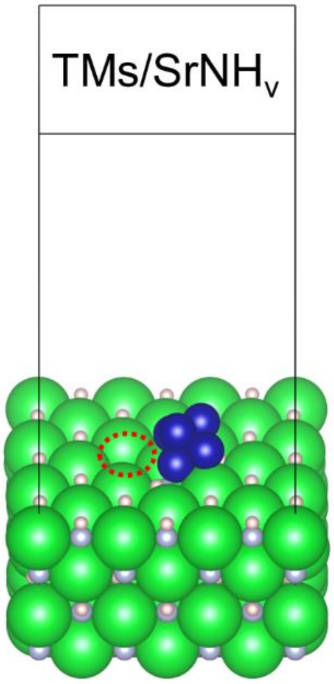

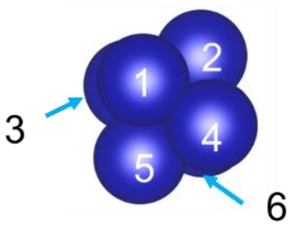

TMs	Co	Fe	Ni
1	-0.15	-0.12	-0.15
2	-0.14	-0.12	-0.12
3	-0.33	-0.33	-0.26
4	-0.34	-0.33	-0.26
5	0.03	0.2	0.11
6	0.07	0.21	0.08
Average	-0.143	-0.082	-0.1

Figure S16 Bader charge analysis of *TMs*/SrNH (*TMs* = Fe, Co, Ni). Calculated Bader charge of Fe, Co, Ni loaded SrNH with surface NH^{2-} vacancies.

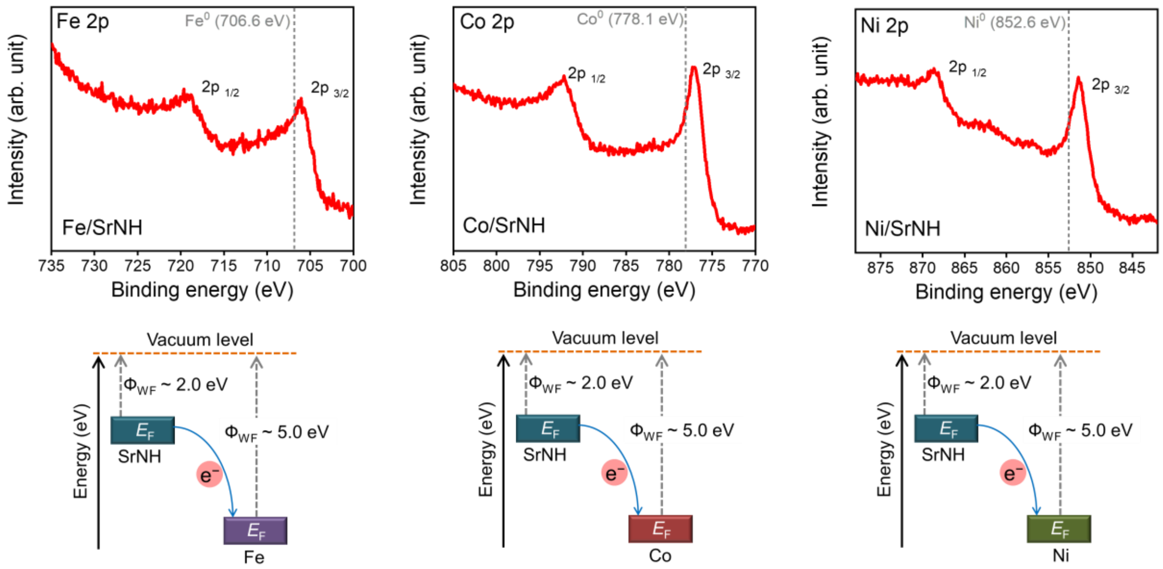

Figure S17 Valence state and electron transfer behavior of TMs on the surface of SrNH. XPS spectra of Fe2p, Co 2p and Ni 2p in TMs-SrNH catalyst. The gray dashed line represents the binding energy of the reference TMs metal with zero valence state. Bottom: comparison of the Fermi level of SrNH and the loaded TMs metal. Blue arrows indicate the direction of electron transfer from SrNH to TMs.

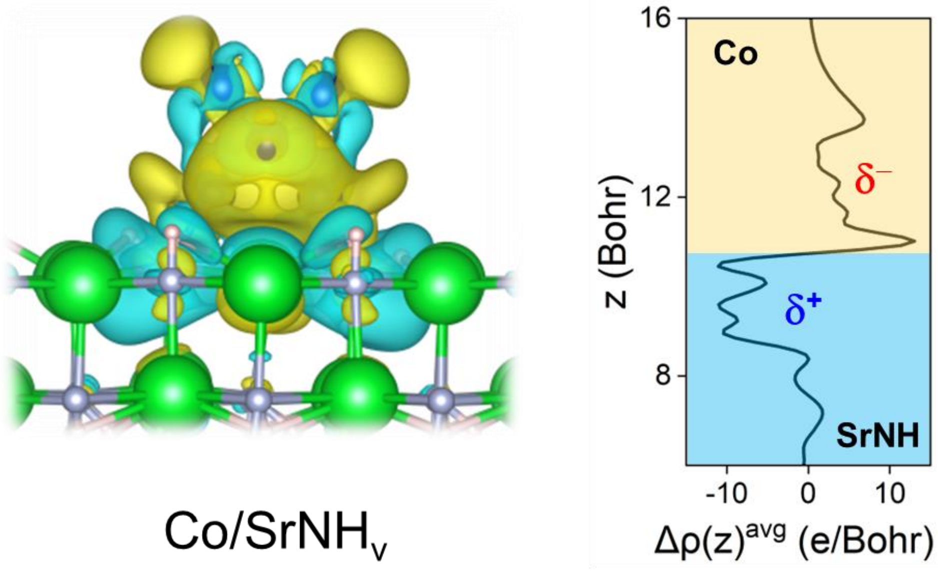

Figure S18 Depictions of the electronic interaction between Co and SrNH. (a) 3D electron density iso-surface map and (b) planar-averaged electron density difference $\Delta\rho$ (z) of Co/SrNH$_v$. The yellow and cyan areas indicate electron accumulation and depletion, respectively.

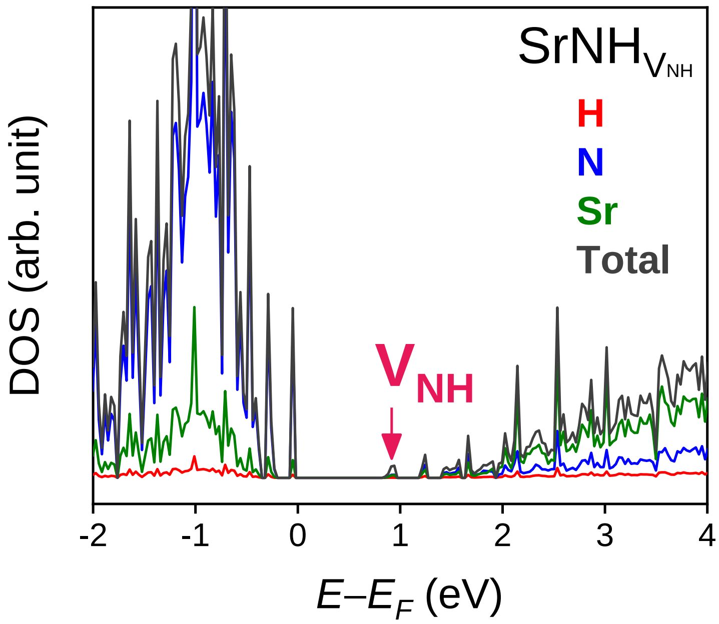

Figure S19 Electronic structure of SrNH$_v$. Projected density of states (DOS) for SrNH with NH^{2-} vacancy.

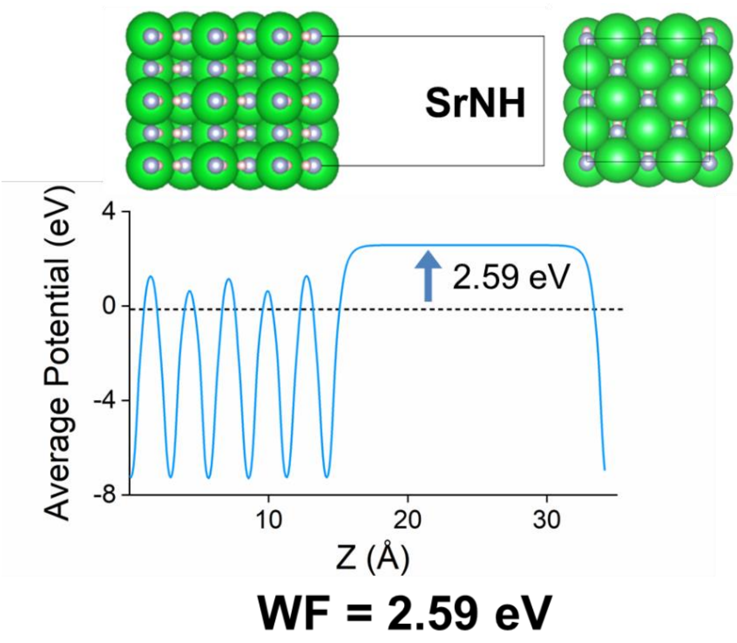

Figure S20 Calculated work function of SrNH.

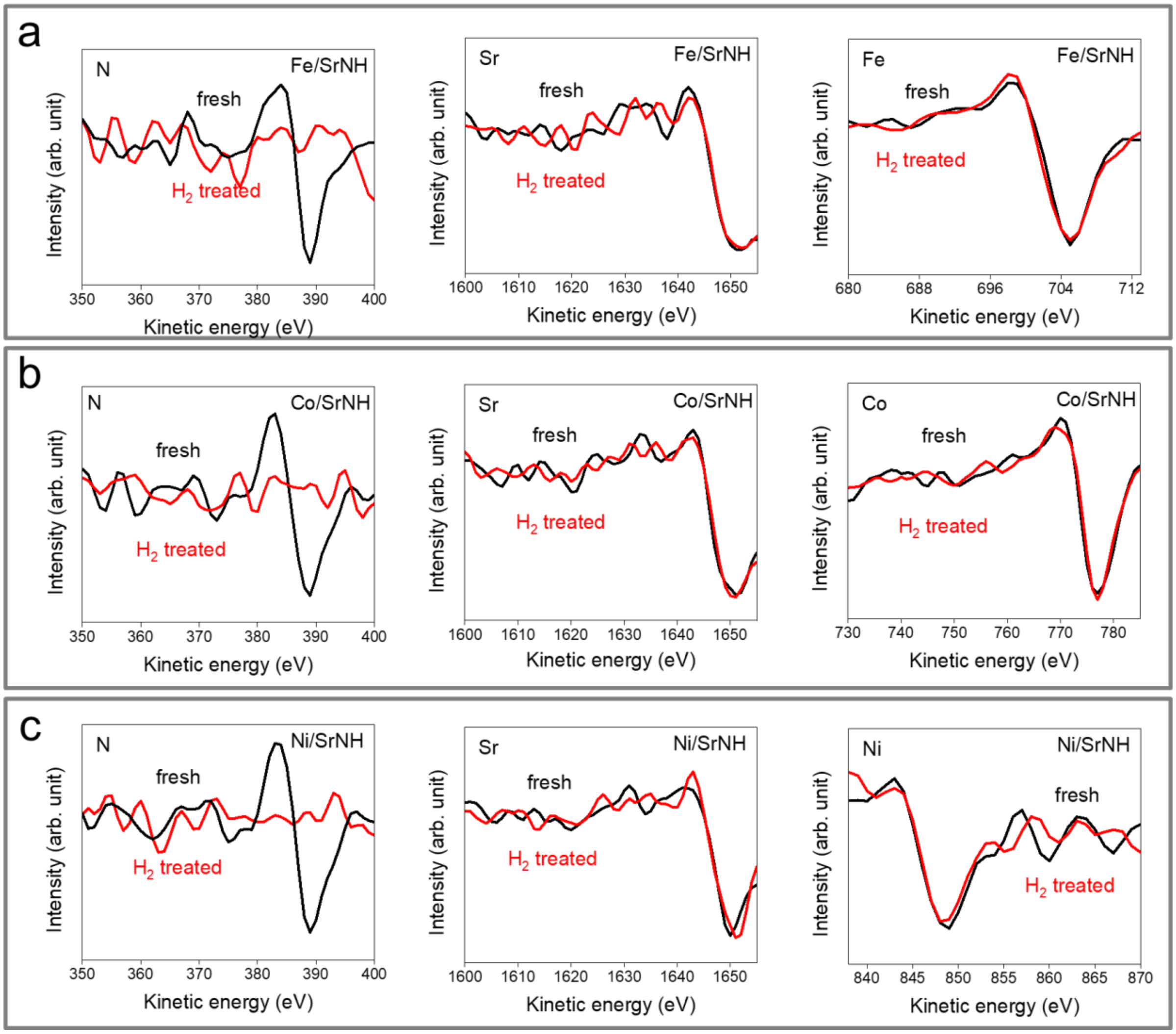

Figure S21 Surface characterization of *TMs*/SrNH (*TMs* = Fe, Co, Ni). AES spectra for N, Sr and TMs of fresh and H_2-treated Fe/SrNH, Co/SrNH and Ni/SrNH catalysts.

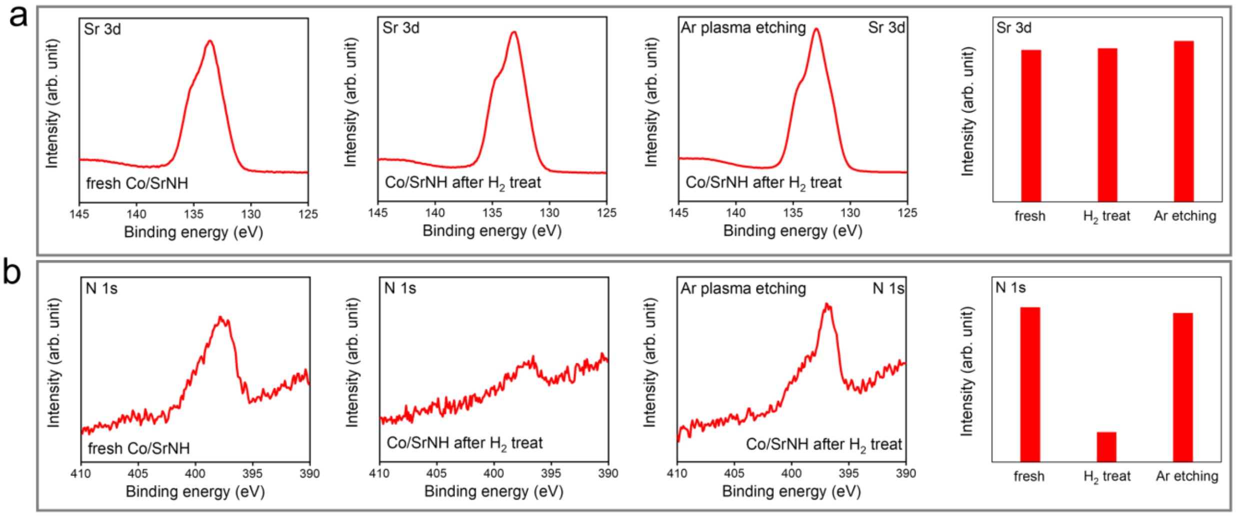

Figure S22 Surface characterization of Co/SrNH. XPS spectra of (a) Sr 3d and (b) N 1s in fresh Co/SrNH, H_2 treated Co/SrNH and with Ar plasma etching.

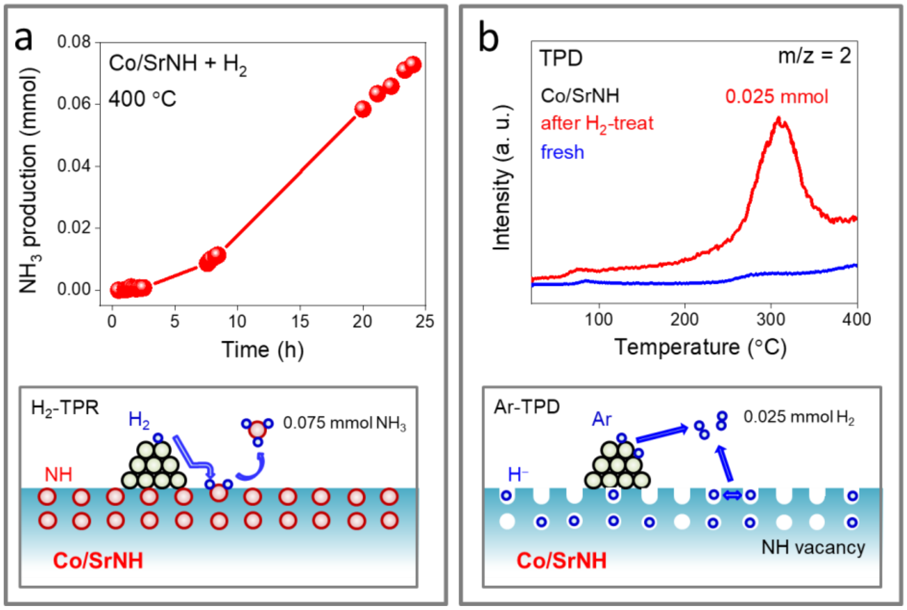

Figure S23 H_2-TPR and TPD measurement over Co/SrNH. (a) Cumulative amount of ammonia generated over Co/SrNH under pure H_2 at 400 °C as a function of reaction time. (b) TPD profile for used Co/SrNH after 24 h of H_2 treatment.

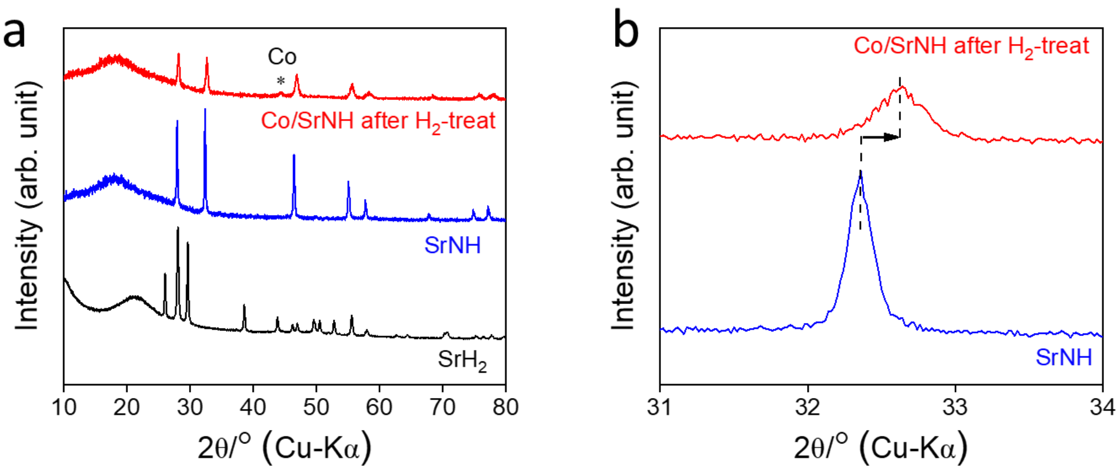

Figure S24 Crystal structure evolution over SrH_2, SrNH and H_2 treated Co/SrNH. (a) Powder XRD patterns for SrH_2, SrNH and H_2 treated Co/SrNH. (b) The enlarged XRD patterns to emphasize the shift of the (200) peak for the corresponding samples. It is also found that the unit cell of H_2-treated sample was slightly shrink, plausibly due to NH vacancy formation and H^- ions incorporation.

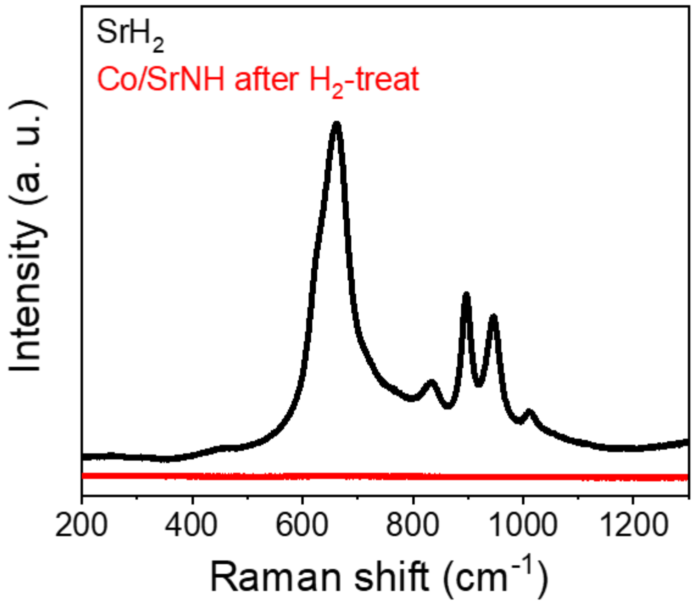

Figure S25 Raman spectra of SrH_2 and H_2 treated Co/SrNH.

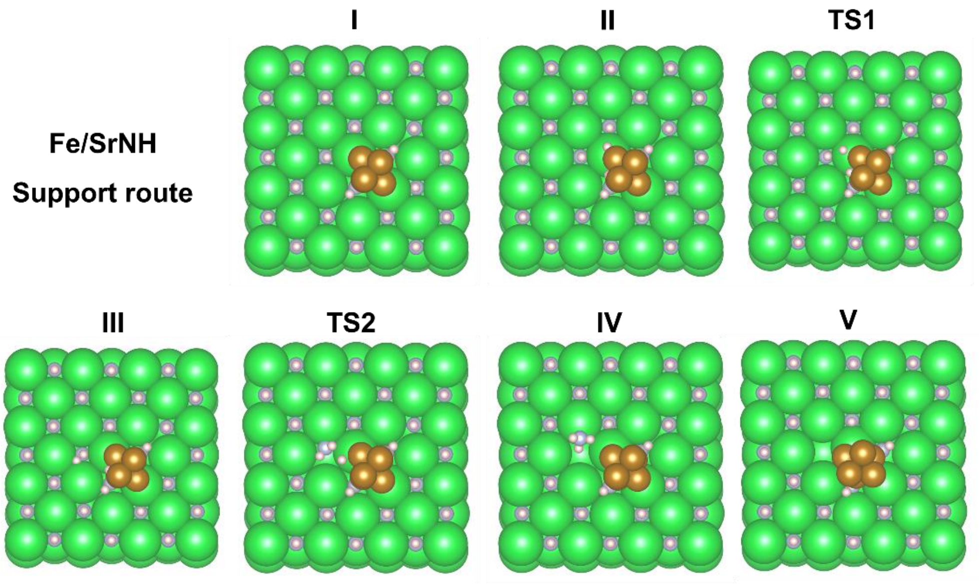

Figure S26 Structures of the intermediates states for the key elementary steps over Fe/SrNH. Proposed reaction pathway for hydrogenation of the lattice NH^{2-} of SrNH support in Fe catalyst.

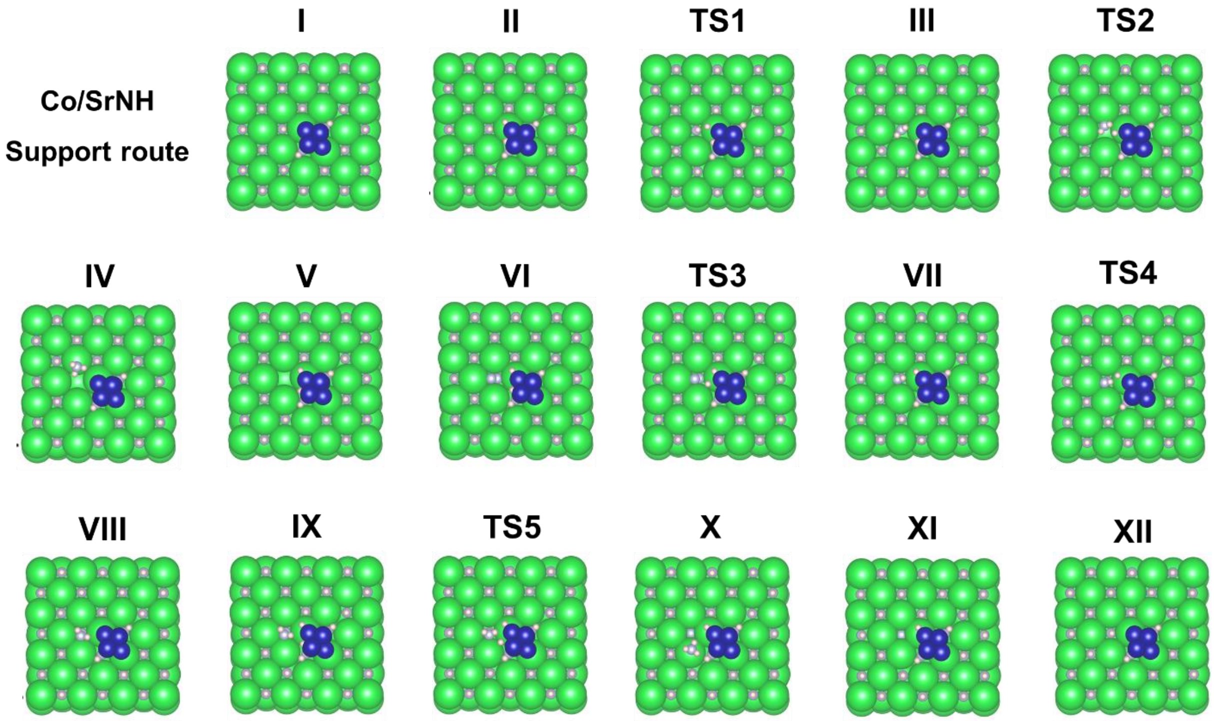

Figure S27 Structures of the intermediates states for the key elementary steps over Co/SrNH. Proposed reaction pathway for hydrogenation of the lattice NH^{2-} of SrNH support in Co catalyst.

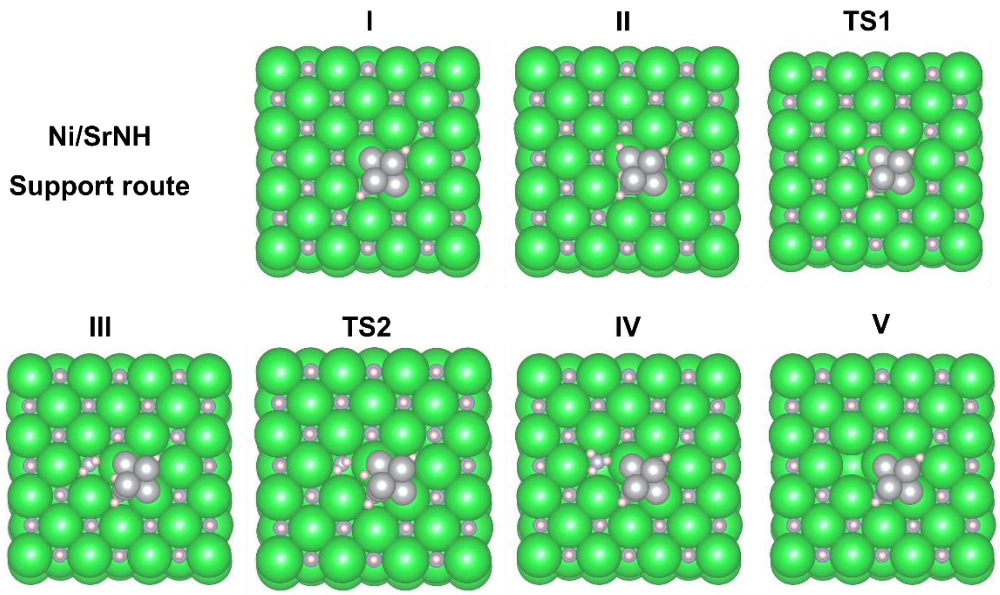

Figure S28 Structures of the intermediates states for the key elementary steps over Ni/SrNH. Proposed reaction pathway for hydrogenation of the lattice NH^{2-} of SrNH support in Ni catalyst.

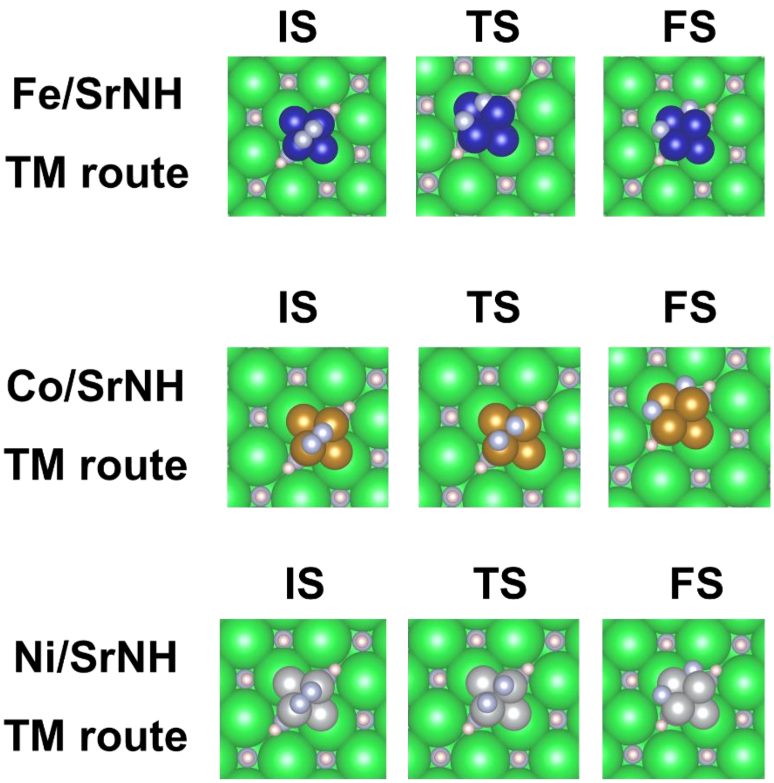

Figure S29 Structures of the intermediates states for the key elementary steps over *TMs*/SrNH (*TMs* = Fe, Co, Ni). Proposed reaction pathway for N_2 dissociation on TMs surface of Fe-, Co-, Ni-SrNH catalysts.

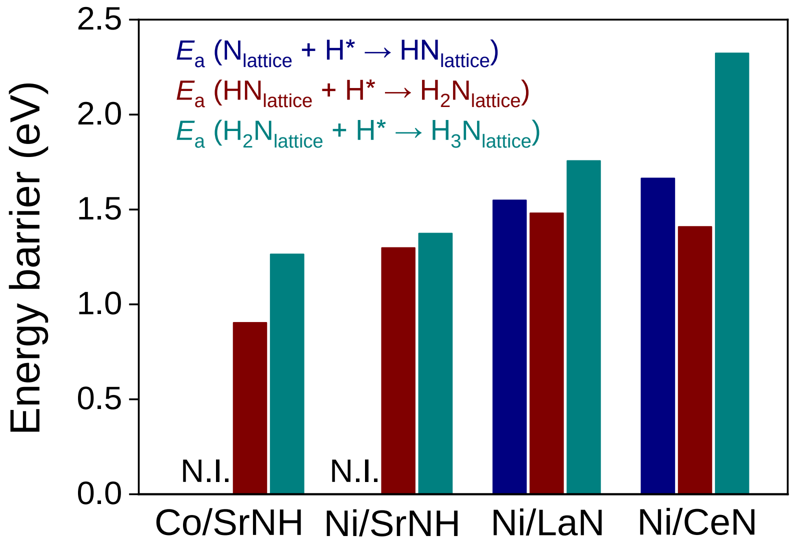

Figure S30 Energy barrier comparison of imides and nitrides catalysts. Calculated energy barrier for NH_x hydrogenation over Co/SrNH, Ni/SrNH, Ni/LaN and Ni/CeN catalysts. N. I. represents not involve.

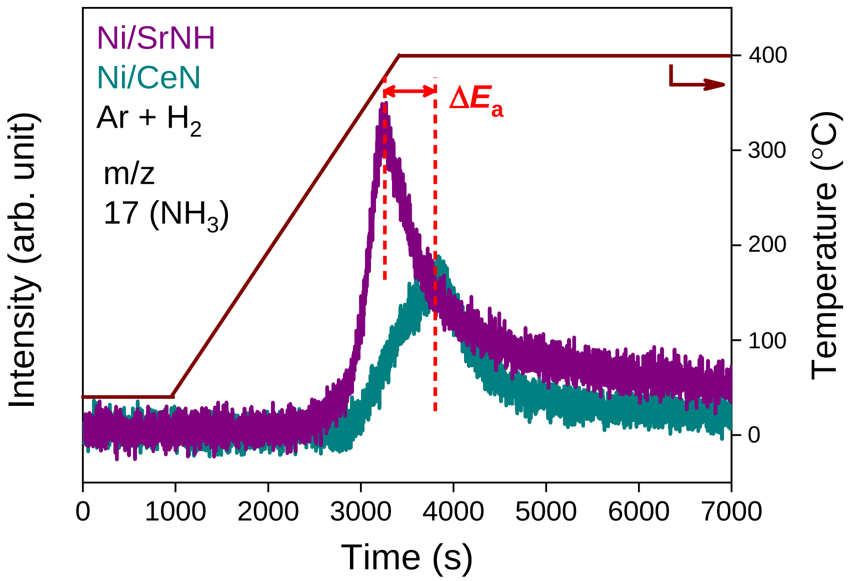

Figure S31 Reaction temperature comparison of SrNH and CeN catalysts. H_2-TPR profiles for Ni/SrNH and Ni/CeN catalysts under Ar and H_2 (20 mL min^{-1} reaction gas, Ar/H_2 = 1:1) at the temperature increased from room temperature to 400 °C.

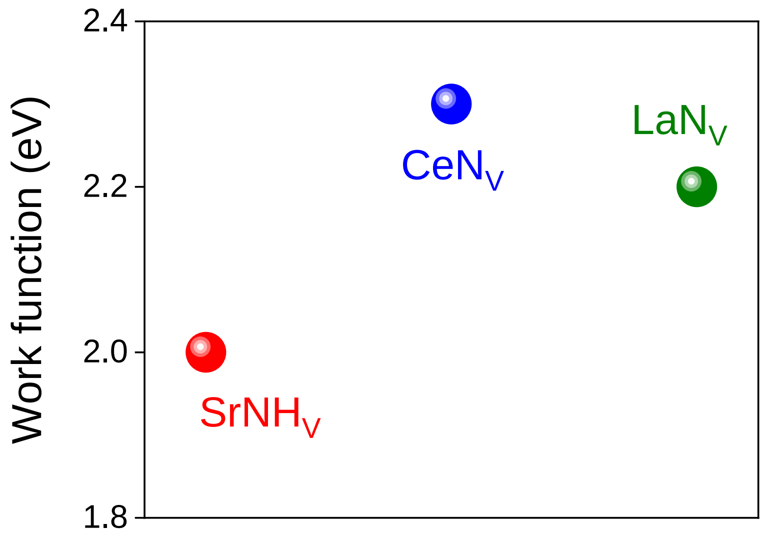

Figure S32 Work function comparison of imide and nitrides catalysts. Calculated work function of defective SrNH, CeN and LaN.

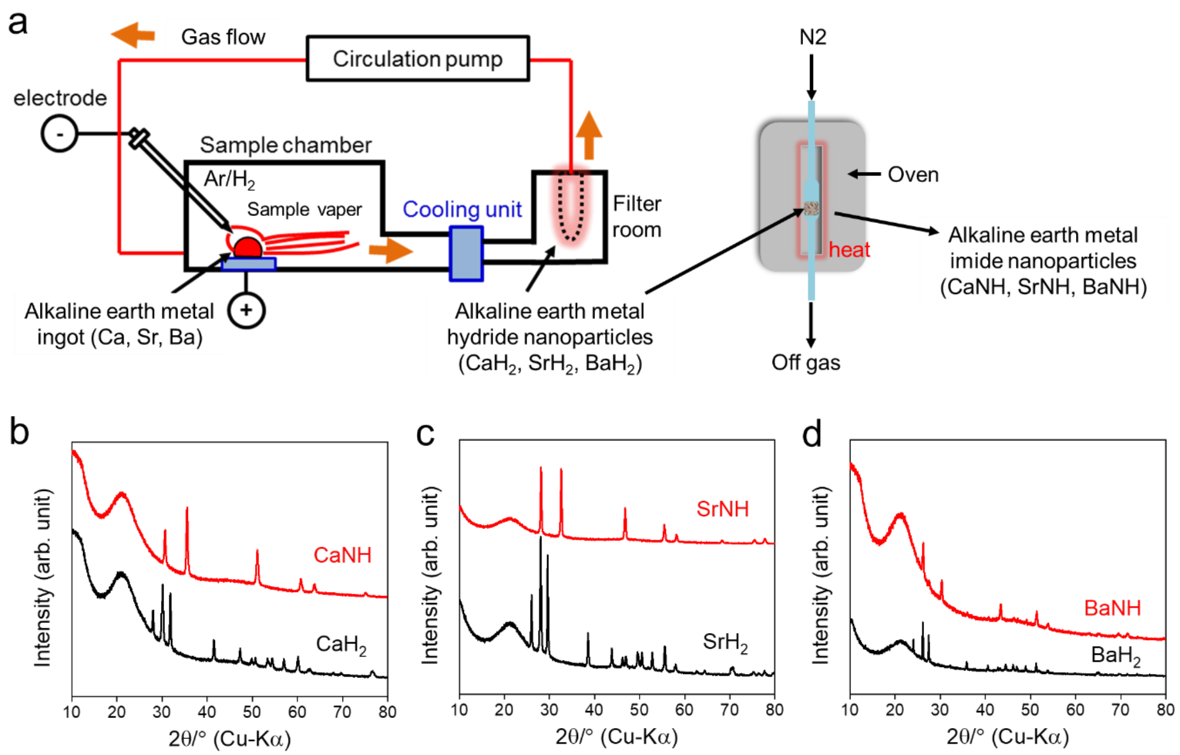

Figure S33 Preparation and crystal structure characterizations of *Ae*NH (*Ae* = Ca, Sr, Ba). (a) Schematic illustration of Ar/H_2 arc evaporation process (left) for the preparation of AeH_2 and N_2 calcination process (right) for fabricating *Ae*NH; Powder XRD patterns for as prepared (b) CaH_2 and CaNH, (c) SrH_2 and SrNH, (d) BaH_2 and BaNH.

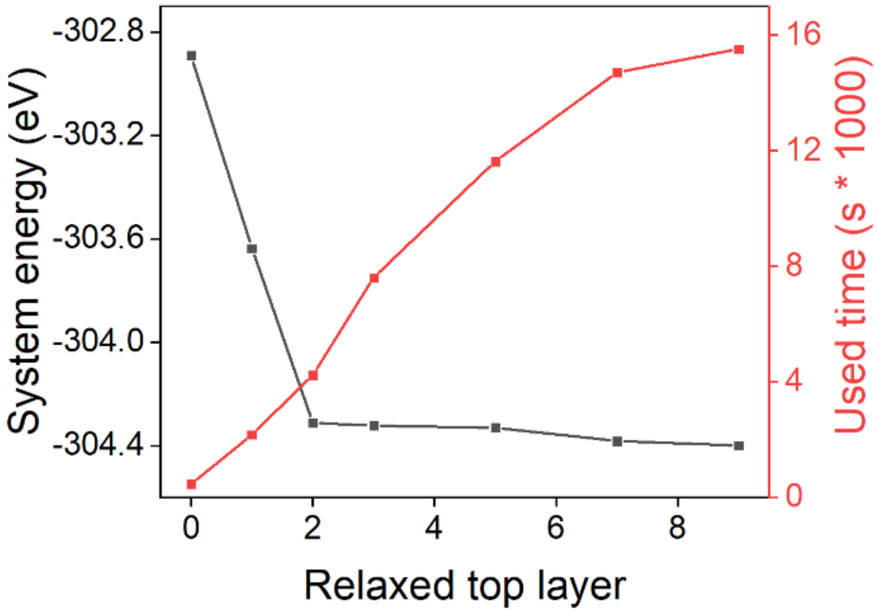

Figure S34 System energies and models used time with different relaxed top layer. Normally, the fixation of the bottom 2-3 layers and relaxation of the top 1-4 layers in model setting is well accepted. Here, the relationship between the relaxed top layers and the systemic accuracy as well as time cost are shown. The time consumption increased monotonically with the relaxed top layers. On the contrary, the system energy continuously decreased over the few relaxed top layers and then remained largely unchanged at more than 2 layers. Considering the both systemic accuracy and time consumption, we chose the 2 top layers for the relaxation in this work.

Table S1 Comparison of the results reported for ammonia synthesis over various Co- and Ni- based catalysts at ambient pressure.

Catalyst	[a] Reaction rate ($mmol \cdot g_{cat}^{-1} \cdot h^{-1}$)	Reaction rate ($mmol \cdot g_{Co}^{-1} \cdot h^{-1}$)	Reaction condition	WHSV ($mL \cdot g^{-1} \cdot h^{-1}$)	Reference
Co/SrNH (4.8 wt% Co)	**7.4**	**154.2**	360 °C, 0.1 MPa	36000	**This work**
	**5.0**	**104.2**	340 °C, 0.1 MPa	36000	**This work**
	**2.4**	**50.0**	300 °C, 0.1 MPa	36000	**This work**
Co/CeN (10.8 wt% Co)	5.76	53.3	360 °C, 0.1 MPa	36000	1
6K−FePc80CoPc20 (20 wt% Co)	~2.5	12.5	400 °C, 0.1 MPa	12000	2
Ni/CeN (11.7 wt% Ni)	3.8	[c]55.6	340 °C, 0.1 MPa	36000	3
Ni/LaN (12.5 wt% Ni)	2.5	[c]44.3	340 °C, 0.1 MPa	36000	4
Co-C-N (3.73 wt% Co)	2.9	77.7	350 °C, 0.2 MPa	60000	5
Co/C12A7:e^- (2.6 wt% Co)	1.7	65.4	400 °C, 0.1 MPa	18000	6
LaCoSi (26.1wt% Co)	1.25	4.8	400 °C, 0.1 MPa	36000	7
Co-Mo/CeO_2 (2.5 wt% Co)	1.5	60.0	460 °C, 0.1 MPa	72000	8
Co-BaH_2 (5.2 wt% Co)	[b]1.8	35.9	300 °C, 0.1 MPa	60000	9
	0.6	11.1	300 °C, 0.1 MPa	60000	
Cs-Co_3Mo_3N (20.5 wt% Co)	~1.0	4.9	400 °C, 0.1 MPa	9000	10

[a] Catalytic process
[b] Chemical Looping process
[c] Reaction rate of $mmol \cdot g_{Ni}^{-1} \cdot h^{-1}$

Table S2 Comparison of results reported for ammonia synthesis over various Co- and Ni- based catalysts at high pressure conditions.

Catalyst	Reaction rate ($mmol \cdot g_{cat}^{-1} \cdot h^{-1}$)	Reaction rate ($mmol \cdot g_{Co}^{-1} \cdot h^{-1}$)	Reaction condition	WHSV ($mL \cdot g^{-1} \cdot h^{-1}$)	Reference
Co/SrNH (4.8 wt% Co)	**37.4**	**779.2**	400 °C, 0.9 MPa	36000	**This work**
	**17.3**	**361.3**	340 °C, 0.9 MPa	36000	**This work**
	**8.8**	**183.3**	300 °C, 0.9 MPa	36000	**This work**
Co/SrNH (1.5 wt% Co)	**25.3**	**1686.7**	400 °C, 0.9 MPa	36000	**This work**
	**12.7**	**846.7**	340 °C, 0.9 MPa	36000	**This work**
	**5.9**	**393.3**	300 °C, 0.9 MPa	36000	**This work**
$Co/BaAl_2O_{4-x}H_y$ (4.7 wt%)	32.5	691.4	400 °C, 0.9 MPa	36000	17
	8.3	176.6	340 °C, 0.9 MPa	36000	
	5.1	108.5	300 °C, 0.9 MPa	36000	
Co/CeN (10.8 wt% Co)	14.2	131.5	340 °C, 0.9 MPa	36000	1
6K−FePc80CoPc20 (20 wt% Co)	3.0	15.0	300 °C, 1.0 MPa	12000	2
Co@BaO/MgO-700 red (20 wt% Co)	24.6	123.0	350 °C, 1.0 MPa	72000	11
LiH/Co-Mg-O (21.3 wt% Co)	19.0	89.2	300 °C, 1.0 MPa	60000	12
Co-C-N (3.73 wt% Co)	4.3	115.3	350 °C, 1.0 MPa	60000	5
Ni/CeN (11.7 wt% Ni)	21.8	[a]186.3	400 °C, 0.9 MPa	36000	3
Ni/LaN (12.5 wt% Ni)	15.5	[a]124.0	400 °C, 0.9 MPa	36000	4
$Co/BaCeO_{3-x}N_yH_z$ (4.7 wt% Co)	2.3	48.9	300 °C, 0.9 MPa	36000	13
$Co/Ba\text{-}Ca(NH_2)_2$ (8 wt% Co)	6.6	82.5	300 °C, 0.9 MPa	36000	14
$Co/C12A7{:}e^-$ (2.6 wt% Co)	3.8	146.2	400 °C, 0.9 MPa	18000	6
LaCoSi (26.1wt% Co)	5.0	19.2	400 °C, 0.9 MPa	36000	7
$Co\text{-}Mo/CeO_2$	1.0	40.0	340 °C, 0.9 MPa	72000	8

(2.5 wt% Co)					
Co-LiH (59.6 wt% Co)	4.7	7.9	300 °C, 1.0 MPa	60000	15
BaH_2-Co/CNTs (5.2 wt% Co)	4.8	92.3	300 °C, 1.0 MPa	60000	16
Cs-Co_3Mo_3N (20.5 wt% Co)	5.0	24.4	400 °C, 1.1 MPa	9000	10

[a] Reaction rate of mmol·g_{Ni}^{-1}·h^{-1}

Table S3. TOFs comparison of the reported Co based catalysts for ammonia synthesis under high pressure conditions.

Catalyst	TOFs (h^{-1})	Reaction condition	WHSV ($mL \cdot g^{-1} \cdot h^{-1}$)	Reference
[a] Co/SrNH (4.8 wt% Co)	**500**	400 °C, 0.9 MPa	36000	**This work**
	**231**	340 °C, 0.9 MPa	36000	**This work**
	**118**	300 °C, 0.9 MPa	36000	**This work**
[a] Co/SrNH (1.5 wt% Co)	**491**	400 °C, 0.9 MPa	36000	**This work**
	**247**	340 °C, 0.9 MPa	36000	**This work**
	**115**	300 °C, 0.9 MPa	36000	**This work**
[b] $Co/BaAl_2O_{4-x}H_y$	205	400 °C, 0.9 MPa	36000	17
(4.7 wt% Co)	97	340 °C, 0.9 MPa	36000	
(1.4 wt% Co)	154	340 °C, 0.9 MPa	36000	
[b] 6K−FePc80CoPc20 (20 wt% Co)	35	300 °C, 1.0 MPa	12000	2
[a] Co/CeN (10.8 wt% Co)	39	340 °C, 0.9 MPa	36000	1
[b] Co@BaO/MgO-700 red (20 wt% Co)	77	350 °C, 1.0 MPa	72000	11
[b] LiH/Co-Mg-O (21.3 wt% Co)	52	300 °C, 1.0 MPa	60000	12
[b] Co-C-N (3.73 wt% Co)	6.7	350 °C, 1.0 MPa	60000	5
[b] Co/C12A7:e^- (2.6 wt% Co)	32	400 °C, 0.9 MPa	18000	6
[c] $Co/BaCeO_{3-x}N_yH_z$ (4.7 wt% Co)	2.89	300 °C, 0.9 MPa	36000	13
[b] $Co/Ba\text{-}Ca(NH_2)_2$ (8 wt% Co)	13	300 °C, 0.9 MPa	36000	14
[c] LaCoSi (26.1wt% Co)	11	400 °C, 0.9 MPa	36000	7

[b] Co-Mo/CeO_2 (2.5 wt% Co)	8.9	340 °C, 0.9 MPa	72000	8
[c] Co-LiH (59.6 wt% Co)	0.47	300 °C, 1.0 MPa	60000	15
[b] BaH_2-Co/CNTs (5.2 wt% Co)	45	300 °C, 1.0 MPa	60000	16
[c] Cs-Co_3Mo_3N (20.5 wt% Co)	1.44	400 °C, 0.1 MPa	9000	10

[a] The TOF was calculated based on the amount of both surface Co sites and surface vacancy sites.

[b] The TOF was calculated based on the amount of surface Co sites.

[c] The TOF was calculated based on the total amount of Co metal.

Table S4 The list of catalysts shown in Figure S13.

Catalyst No.	Catalyst	Reference
1	$Co/BaAl_2O_{4-x}H_y$ (4.7 wt% Co)	*J. Am. Chem. Soc.* **145**, 10669−10680 (2023).
2	$Co/BaAl_2O_{4-x}H_y$ (1.4 wt% Co)	*J. Am. Chem. Soc.* **145**, 10669−10680 (2023).
3	6K-FePc80CoPc20 (20 wt% Co)	*ACS Catal.* **12,** 587–599 (2022).
4	Co/CeN (10.8 wt% Co)	*J. Am. Chem. Soc.* **143**, 12857–12866 (2021).
5	Co@BaO/MgO-700 red (20 wt% Co)	*ACS Catal*. **11**, 13050–13061 (2021).
6	LiH/Co-Mg-O (21.3 wt% Co)	*Chem. Commun*. **57**, 8576–8579 (2021).
7	Co-C-N (3.73 wt% Co)	*Nat. Commun*. **11**, 653 (2020).
8	Co/C12A7:e^- (2.6 wt% Co)	*ACS Catal.* **9**, 1670–1679 (2019).
9	$Co/BaCeO_{3-x}N_yH_z$ (4.7 wt% Co)	*J. Am. Chem. Soc*. **141**, 20344–20353 (2019).
10	$Co/Ba\text{-}Ca(NH_2)_2$ (8 wt% Co)	*Angew. Chem. Int. Ed.* **57**, 2648–2652 (2018).
11	LaCoSi (26.1 wt% Co)	*Nat. Catal.* **1**, 178–185 (2018).
12	$Co\text{-}Mo/CeO_2$ (2.5 wt% Co)	*J. Catal*. **364**, 31–39 (2018).
13	Co-LiH (59.6 wt% Co)	*Nat. Chem.* 9, 64–70 (2017).
14	$BaH_2\text{-}Co/CNTs$ (5.2 wt% Co)	*ACS Catal*. 7, 3654–3661 (2017).
15	$Cs\text{-}Co_3Mo_3N$ (20.5 wt% Co)	*Appl. Catal. A-Gen.* 218, 121–128 (2001).
16	$Ru/BaAl_2O_{4-x}H_y$ (~2 wt% Ru)	*J. Am. Chem. Soc*. **145**, 10669−10680 (2023).
17	$Ru/BaCeO_{3-x}N_yH_z$ (4.5 wt% Ru)	*J. Am. Chem. Soc*. **141,** 20344−20353 (2019)
18	LaRuSi (37.7 wt% Ru)	*Angew. Chem. Int. Ed.* **58**. 825−829 (2019)
19	$Ru/BaTiO_{2.5}H_{0.5}$ (5 wt% Ru)	*Adv. Energy Mater*. **8**, 1801772 (2018).
20	Cs−Ru/MgO (10 wt% Ru)	*Angew. Chem. Int. Ed.* **57**, 2648−2652 (2018).
21	$Ru/Ba\text{−}Ca(NH_2)_2$ (10 wt% Ru)	*Angew. Chem. Int. Ed.* **57**, 2648−2652 (2018).
22	$Ru/La_{0.5}Ce_{0.5}O_{1.75}$(5 wt% Ru)	*Chem. Sci.* **9**, 2230−2237 (2018).
23	$Ru/Ba\text{−}Ca(NH_2)_2$ (10 wt% Ru)	*ACS Catal*. **8**, 10977−10984 (2018).
24	Ru/Pr_2O_3 (5 wt % Ru)	*Chem. Sci*. **8**, 674–679 (2017).

Table S5. TOFs comparison of the reported Ru based catalysts for ammonia synthesis under high pressure conditions.

Catalyst	TOF (h^{-1})	Reaction condition	WHSV ($mL\cdot g^{-1}\cdot h^{-1}$)	Reference
[a] Co/SrNH (4.8 wt% Co)	**500**	400 °C, 0.9 MPa	36000	**This work**
	**231**	340 °C, 0.9 MPa	36000	**This work**
	**118**	300 °C, 0.9 MPa	36000	**This work**
[a] Co/SrNH (1.5 wt% Co)	**491**	400 °C, 0.9 MPa	36000	**This work**
	**247**	340 °C, 0.9 MPa	36000	**This work**
	**115**	300 °C, 0.9 MPa	36000	**This work**
[b] Ru/$BaAl_2O_{4-x}H_y$ (~2 wt% Ru)	205	340 °C, 0.9 MPa	36000	17
[c] Ru/$BaCeO_{3-x}N_yH$ (4.5 wt% Ru)	63	400 °C, 0.9 MPa	36000	13
[c] LaRuSi (37.7 wt% Ru)	1.2	400 °C, 0.9 MPa	36000	18
[b] Ru/$BaTiO_{2.5}H_{0.5}$ (5 wt% Ru)	112	400 °C, 1 MPa	66000	19
[b] Cs–Ru/MgO (10 wt% Ru)	1.4	300 °C, 0.9 MPa	36000	20
[b] Ru/Ba–$Ca(NH_2)_2$ (10 wt% Ru)	48	300 °C, 0.9 MPa	36000	20
[b] Ru/$La_{0.5}Ce_{0.5}O_{1.75}$(5 wt% Ru)	183	350 °C, 1 MPa	72000	21
[b] Ru/BaO–CaH_2 (10 wt% Ru)	52	340 °C, 0.9 MPa	36000	22
[b] Ru/Pr_2O_3 (5 wt % Ru)	180	390 °C, 0.9 MPa	18000	23

[a] The TOF was calculated based on the amount of both surface Co sites and surface vacancy sites.

[b] The TOF was calculated based on the amount of surface Ru sites.

[c] The TOF was calculated based on total amount of Ru metal.

Table S6 Intrinsic and reactive formation energy of the vacancy.

Sample	Intrinsic (eV)	With H help (eV)
SrO	5.38	2.92

Table S7 Formation energy of three types of vacancy.

type of vacancy	Formation energy (eV)
V_{NH}	1.92
N_{NH}	2.48
H_{NH}	1.17

Table S8 DFT calculated energy changes for various reaction steps over Co/SrNH for ammonia synthesis

Step	Elementary reaction steps	*E* (eV)	Step	TS (eV)
II	$NH_{lattice} + H_2(g) \rightarrow 2H^*$	-1.427		---
III	$NH_{lattice} + H^* \rightarrow HN_{2lattice}$	-0.611	II → III	-0.523
IV	$NH_{2lattice} + H^* \rightarrow NH_3$	0.465	III → IV	0.653
V	$NH_3 + H_2(g) \rightarrow NH_3(g) + V_{NH} + 2H^*$	-0.191	IV → V	---
VI	$V_{NH} + N_2(g) \rightarrow N_2$	-0.981	V → VI	---
VII	$N_2 + H^* \rightarrow N_2H$	-0.920	VI → VII	-0.496
VIII	$N_2H + H^* \rightarrow N_2H_2$	-0.437	VII → VIII	-0.141
IX	$N_2H_2 + H_2(g) \rightarrow N_2H_2 + 2H^*$	-1.846	VIII→ IX	---
X	$N_2H_2 + H^* \rightarrow N_2H_3$	-1.764	IX → X	-0.755
XI	$N_2H_3 \rightarrow N_{lattice} + NH_3(g)$	-1.517	X → XI	---
XII	$N_{lattice} + H^* \rightarrow NH_{lattice}$	-2.158	XI → XII	---

Table S9 DFT calculated energy changes for various reaction steps over Fe/SrNH and Ni/SrNH for ammonia synthesis

Step	Elementary reaction steps	*E* (eV)	Step	TS (eV)
[a]II	$NH_{lattice} + H_2 (g) \rightarrow 2H^*$	-1.398		---
[a]III	$NH_{lattice} + H^* \rightarrow HN_{2lattice}$	-0.308	II → III	0.019
[a]IV	$NH_{2lattice} + H^* \rightarrow NH_3$	0.630	III → IV	1.351
[a]V	$NH_3 + H_2 (g) \rightarrow NH_3 (g) + V_{NH} + 2H^*$	-0.159	IV → V	---
[b]II	$NH_{lattice} + H_2 (g) \rightarrow 2H^*$	-1.464		---
[b]III	$NH_{lattice} + H^* \rightarrow HN_{2lattice}$	-0.576	II → III	-0.166
[b]IV	$NH_{2lattice} + H^* \rightarrow NH_3$	0.614	III → IV	0.798
[b]V	$NH_3 + H_2 (g) \rightarrow NH_3 (g) + V_{NH} + 2H^*$	-0.174	IV → V	---

[a] Fe/SrNH
[b] Ni/SrNH

Table S10 DFT calculated energy changes for N_2 dissociation over Fe, Co and Ni of TMs/SrNH catalysts.

Step	Elementary reaction steps	*E* (eV)	Step	TS (eV)
[a]II	$N_2 (g) \rightarrow N_2*$	-1.055		---
[a]III	$N_2* \rightarrow 2N*$	-1.617	II → III	0.135
[b]II	$N_2 (g) \rightarrow N_2*$	-0.856		---
[b]III	$N_2* \rightarrow 2N*$	-0.885	II → III	0.500
[c]II	$N_2 (g) \rightarrow N_2*$	-0.637		---
[c]III	$N_2* \rightarrow 2N*$	-0.287	II → III	1.581

[a] Fe/SrNH
[b] Co/SrNH
[c] Ni/SrNH

Table S11 Surface energy of SrNH.

Crystal index	Surface energy (J/m^2)
0 0 1	0.762
1 1 0	1.362
1 1 1	3.644

Table S12 System energy of Co cluster loading on different sites of SrNH.

Cluster sites	System energy (eV)
NH site	-1128.856
Sr site	-1128.735

Table S13 Intrinsic and reactive formation energy of the NH vacancy.

AeNH	Intrinsic (eV)	With H help (eV)
CaNH	2.35	1.88
SrNH	1.92	1.45
BaNH	0.85	0.38

Supplementary References

1. Ye, T. N., *et al.* Dissociative and associative concerted mechanism for ammonia synthesis over Co-based catalyst. *J. Am. Chem. Soc.* **143**, 12857-12866 (2021).

2. Rai, R. K., *et al.* Iron-cobalt-based materials: an efficient bimetallic catalyst for ammonia synthesis at low temperatures. *ACS Catal.* **12**, 587-599 (2022).

3. Ye, T. N., *et al.* Contribution of nitrogen vacancies to ammonia synthesis over metal nitride catalysts. *J. Am. Chem. Soc.* **142**, 14374-14383 (2020).

4. Ye, T. N., *et al.* Vacancy-enabled N_2 activation for ammonia synthesis on an Ni-loaded catalyst. *Nature* **583**, 391-395 (2020).

5. Wang, X., *et al.* Insight into dynamic and steady-state active sites for nitrogen activation to ammonia by cobalt-based catalyst. *Nat. Commun.* **11**, 653 (2020).

6. Inoue, Y., *et al.* Direct activation of cobalt catalyst by $12CaO\cdot 7Al_2O_3$ electride for ammonia synthesis. *ACS Catal.* **9**, 1670-1679 (2019).

7. Gong, Y., *et al.* Ternary intermetallic LaCoSi as a catalyst for N_2 activation. *Nat. Catal.* **1**, 178-185 (2018).

8. Tsuji, Y., *et al.* Control of nitrogen activation ability by Co-Mo bimetallic nanoparticle catalysts prepared via sodium naphthalenide-reduction. *J. Catal.* **364**, 31-39 (2018).

9. Gao, W., *et al.* Production of ammonia via a chemical looping process based on metal imides as nitrogen carriers. *Nat. Energy* **3**, 1067-1075 (2018).

10. Kojima, R. & Aika, K-i. Cobalt molybdenum bimetallic nitride catalysts for ammonia synthesis part 2. kinetic study. *Appl. Catal. A-Gen.* **218**, 121-128 (2001).

11. Sato, K., *et al.* Barium oxide encapsulating cobalt nanoparticles supported on magnesium oxide: active non-noble metal catalysts for ammonia synthesis under mild reaction conditions. *ACS Catal.* **11**, 13050-13061 (2021).

12. Gao, W., *et al.* In situ formed Co from a Co-Mg-O solid solution synergizing with LiH for efficient ammonia synthesis. *Chem. Commun.* **57**, 8576-8579 (2021).

13. Kitano, M., *et al.* Low-temperature synthesis of perovskite oxynitride-hydrides as ammonia synthesis catalysts. *J. Am. Chem. Soc.* **141**, 20344-20353 (2019).

14. Kitano, M., *et al.* Self-organized ruthenium-barium core-shell nanoparticles on a mesoporous calcium amide matrix for efficient low-temperature ammonia synthesis. *Angew. Chem. Int. Ed.* **57**, 2648-2652 (2018).

15. Wang, P., *et al.* Breaking scaling relations to achieve low-temperature ammonia synthesis through LiH-mediated nitrogen transfer and hydrogenation. *Nat. Chem.* **9**, 64-70 (2017).

16. Gao, W., *et al.* Barium hydride-mediated nitrogen transfer and hydrogenation for ammonia synthesis: a case study of cobalt. *ACS Catal.* **7**, 3654-3661 (2017).

17. Jiang, Y., *et al.* Boosted Activity of Cobalt Catalysts for Ammonia Synthesis with $BaAl_2O_{4-x}H_y$ Electrides. *J. Am. Chem. Soc.* **19**, 10669–10680 (2023).

18. Wu, J., *et al*. Intermetallic Electride Catalyst as a Platform for Ammonia Synthesis. *Angew. Chem. Int. Ed.* **58**, 825−829 (2019)

19. Tang, Y., *et al*. Metal-Dependent Support Effects of Oxyhydride-Supported Ru, Fe, Co Catalysts for Ammonia Synthesis. *Adv. Energy Mater.* **8**, 1801772 (2018).

20. Kitano, M., *et al.* Self-organized Ruthenium–Barium Core–Shell Nanoparticles on a Mesoporous Calcium Amide Matrix for Efficient Low-Temperature Ammonia Synthesis. *Angew. Chem. Int. Ed.* **57**, 2648−2652 (2018).

21. Ogura, Y., *et al.* Efficient ammonia synthesis over a $Ru/La_{0.5}Ce_{0.5}O_{1.75}$ catalyst pre-reduced at high temperature. *Chem. Sci.* **9**, 2230−2237 (2018).

22. Hattori, M., *et al.* Enhanced Catalytic Ammonia Synthesis with Transformed BaO. *ACS Catal.* **8**, 10977−10984 (2018).

23. Sato, K., *et al.* A low-crystalline ruthenium nano-layer supported on praseodymium oxide as an active catalyst for ammonia synthesis. *Chem. Sci.* **8**, 674–679 (2017).
